# Alternative strategies of nutrient acquisition and energy conservation map to the biogeography of marine ammonia-oxidizing archaea

**DOI:** 10.1038/s41396-020-0710-7

**Published:** 2020-07-07

**Authors:** Wei Qin, Yue Zheng, Feng Zhao, Yulin Wang, Hidetoshi Urakawa, Willm Martens-Habbena, Haodong Liu, Xiaowu Huang, Xinxu Zhang, Tatsunori Nakagawa, Daniel R. Mende, Annette Bollmann, Baozhan Wang, Yao Zhang, Shady A. Amin, Jeppe L. Nielsen, Koji Mori, Reiji Takahashi, E. Virginia Armbrust, Mari-K.H. Winkler, Edward F. DeLong, Meng Li, Po-Heng Lee, Jizhong Zhou, Chuanlun Zhang, Tong Zhang, David A. Stahl, Anitra E. Ingalls

**Affiliations:** 1 School of Oceanography, University of Washington, Seattle, WA USA; 2 CAS Key Laboratory of Urban Pollutant Conversion, Institute of Urban Environment, Chinese Academy of Sciences, Xiamen, China; 3 University of Chinese Academy of Sciences, Beijing, China; 4 Environmental Microbiome Engineering and Biotechnology Laboratory, Center for Environmental Engineering Research, Department of Civil Engineering, The University of Hong Kong, Hong Kong, China; 5 Department of Ecology and Environmental Studies, Florida Gulf Coast University, Fort Myers, FL USA; 6 Fort Lauderdale Research and Education Center, Department of Microbiology and Cell Science, Institute of Food and Agricultural Sciences, University of Florida, Davie, FL USA; 7 Department of Ocean Science and Engineering, Shenzhen Key Laboratory of Marine Archaea Geo-Omics, Southern University of Science and Technology, Shenzhen, China; 8 Department of Civil and Environmental Engineering, The Hong Kong Polytechnic University, Hong Kong, China; 9 Shenzhen Key Laboratory of Marine Microbiome Engineering, Institute for Advanced Study, Shenzhen University, Shenzhen, China; 10 College of Bioresource Sciences, Nihon University, Fujisawa, Kanagawa Japan; 11 Daniel K. Inouye Center for Microbial Oceanography: Research and Education, University of Hawaii, Honolulu, HI USA; 12 Department of Microbiology, Miami University, Oxford, OH USA; 13 Key Lab of Microbiology for Agricultural Environment, Ministry of Agriculture, College of Life Sciences, Nanjing Agricultural University, Nanjing, China; 14 State Key Laboratory of Marine Environmental Sciences and College of Ocean and Earth Sciences, Xiamen University, Xiamen, China; 15 Department of Biology, New York University Abu Dhabi, Abu Dhabi, UAE; 16 Department of Chemistry and Bioscience, Aalborg University, Aalborg, Denmark; 17 NITE Biological Resource Center (NBRC), National Institute of Technology and Evaluation (NITE), Kisarazu, Chiba Japan; 18 Department of Civil and Environmental Engineering, University of Washington, Seattle, WA USA; 19 Department of Civil and Environmental Engineering, Imperial College London, London, UK; 20 Institute for Environmental Genomics, Department of Microbiology and Plant Biology, and School of Civil Engineering and Environmental Sciences, University of Oklahoma, Norman, OK USA; 21 Earth and Environmental Sciences, Lawrence Berkeley National Laboratory, Berkeley, CA USA; 22 School of Environment, Tsinghua University, Beijing, China

**Keywords:** Microbial ecology, Water microbiology

## Abstract

Ammonia-oxidizing archaea (AOA) are among the most abundant and ubiquitous microorganisms in the ocean, exerting primary control on nitrification and nitrogen oxides emission. Although united by a common physiology of chemoautotrophic growth on ammonia, a corresponding high genomic and habitat variability suggests tremendous adaptive capacity. Here, we compared 44 diverse AOA genomes, 37 from species cultivated from samples collected across diverse geographic locations and seven assembled from metagenomic sequences from the mesopelagic to hadopelagic zones of the deep ocean. Comparative analysis identified seven major marine AOA genotypic groups having gene content correlated with their distinctive biogeographies. Phosphorus and ammonia availabilities as well as hydrostatic pressure were identified as selective forces driving marine AOA genotypic and gene content variability in different oceanic regions. Notably, AOA methylphosphonate biosynthetic genes span diverse oceanic provinces, reinforcing their importance for methane production in the ocean. Together, our combined comparative physiological, genomic, and metagenomic analyses provide a comprehensive view of the biogeography of globally abundant AOA and their adaptive radiation into a vast range of marine and terrestrial habitats.

## Introduction

The ammonia-oxidizing archaea (AOA) comprise one of the most abundant and ubiquitous groups of microorganisms in the global biosphere. Studies of their distribution and diversity based on sequence variation of single-copy core genes, such as those coding for the 16S rRNA and AmoA (the alpha subunit of ammonia monooxygenase), have shown they span phylum-level diversity and constitute up to 30% of microbial plankton in the oceans and up to 5% of microbial populations in soil [1–7]. Genome sequencing of uncultivated AOA also provided early environmental and genomic perspective. For example, the sponge symbiont *Candidatus* (*Ca.*) Cenarchaeum symbiosum was the first genome sequenced from AOA, yielding new perspective on the evolution, metabolic potential, and environmental distribution of this ubiquitous archaeal lineage [8]. Their habitat range encompasses the oceanic water column from its surface to hadal depths [3, 9], polar oceans [10], symbiotic systems [11], and terrestrial systems of extremes of pH, temperature, and elevation [12–15]. They are now recognized to exert the primary control on nitrification in oligotrophic environments [16–18]. In addition to having a dominant role in the global nitrogen cycle, AOA make a significant contribution to carbon fixation through chemosynthetic pathways [19, 20], the production of greenhouse gases nitrous oxide and methane [21, 22], and the provision of the often limiting cofactor cobalamin (vitamin B_12_) in natural systems [23, 24].

Since the isolation of the first AOA species *Nitrosopumilus maritimus* in 2005 [25], over a decade of worldwide cultivation efforts have resulted in nearly 40 AOA strains enriched or isolated from natural and engineered ecosystems. These cultured species represent an environmentally and phylogenetically diverse group of archaeal ammonia oxidizers, spanning nearly 30% 16S rRNA gene divergence and covering four distinct phylogenetic lineages of the phylum *Thaumarchaeota* [26, 27]. Isolates now in culture provide a basis for investigations of the interrelationships between genotype, physiology, and ecology of this biogeochemically significant group, and for further characterizing the supporting biochemistry. For instance, the integrated genomic, physiological, and biochemical characterizations of the model AOA *Nitrosopumilus maritimus* revealed that the remarkable ecological success of AOA in marine environments is associated with their exceptionally high substrate affinity [17], energy-efficient carbon fixation [19], copper-centric respiratory system [28, 29], and unique cell envelop structure [26, 30–33]. In addition, comparative genomic analysis of three *Nitrososphaera* species and four *Ca*. Nitrosotalea species offered insights in the general adaptive strategies of soil AOA to neutral and low pH environments [34, 35].

In order to develop a better understanding of the adaptive radiation of this globally abundant group, we conducted a comprehensive analysis spanning a broad representation of marine, freshwater, soil, and geothermal AOA species. We framed our comparative analyses using genome sequences from isolates and enrichments from distinct habitats available in the literature and from newly generated genome sequences from our laboratories. Gene content of cultured species was then related to gene content and inferred metabolic traits derived from analysis of global ocean metagenomic databases across four oceans and two seas, spanning from epipelagic to hadopelagic zones. Robust association between variation in gene content among oceanic populations and environmental variables revealed the importance of ammonia and phosphorous availability, as well as hydrostatic pressure, in controlling the biogeography of different AOA genotypes. Niche boundaries were in part defined by the repertoire of genes for ammonia and phosphorous acquisition, and mechanisms of ATP generation. In addition, these analyses identified an extensive and variable reservoir of gene functions that appear to confer locally adaptive traits to marine and terrestrial AOA.

## Materials and methods

### Culture maintenance and genome sequencing

All AOA species were maintained in liquid mineral medium, and their genomic DNA was sequenced by Illumina or Pacbio platforms. For details, see Supplementary information.

### Sampling sites and metagenome-assembled genomes (MAGs)

AOA MAGs were recovered from wasterwater treatment plants, geothermal environments, and hadopelagic waters. Metagenome binning and taxonomic assignments are described in Supplementary information.

### Comparative genomic analysis

All methods for genomic feature, phylogenomic, core genome, and pan-genome analyses of genomes from cultured AOA, MAGs, and single-cell amplified genomes (SAGs) are described in detail in Supplementary information.

### Evolution experiment and mutation analysis

*Nitrosopumilus maritimus* strain SCM1 has been continuously transferred under optimum growth conditions from 2007 to 2018. Cultures were harvested in May 2011 and June 2016 for genome re-sequencing and subsequent mutation analysis. Further details of the methods are given in Supplementary information.

### Distribution and diversity of marine AOA genotypic groups and functional genes in the global ocean

Twenty-five marine AOA species genomes were clustered in seven genotypic groups to represent populations from distinct phylogenetic lineages and ecological habitats. To investigate the overall distribution of these genotypic groups in the global ocean, competitive fragment recruitment was conducted to determine the relative recruitment to available marine AOA species genomes in GOS and *Tara* Oceans metagenomic databases, as well as the metagenomic datasets of the HOT time-series station (125–4000 m), Northeast Pacific Ocean (2000 m), the Yap Trench (5000–5700 m), and the Mariana Trench waters (2000–8000 m). For details, see Supplementary information.

## Results and discussion

**Fig. 1:**
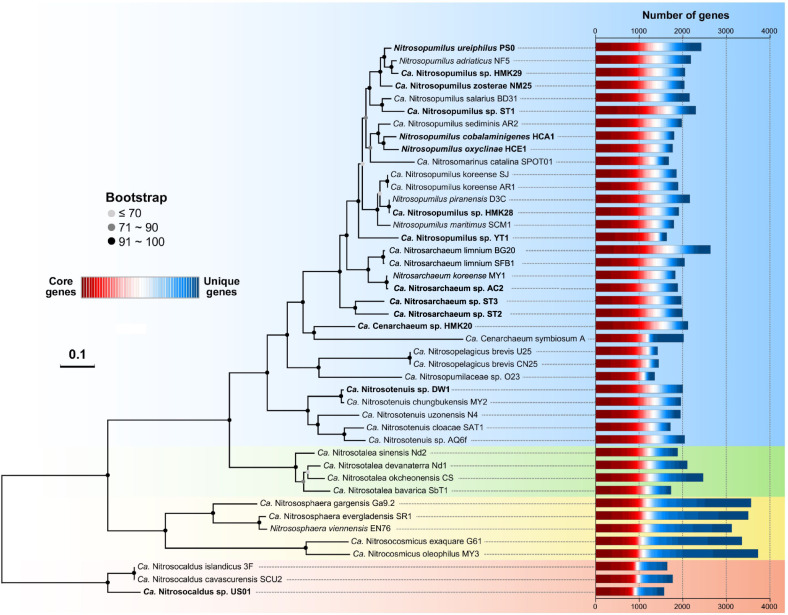
Phylogenomic inference of AOA species affiliated to the orders *Nitrosopumilales* (blue), *Ca*. Nitrosotaleales (green), *Nitrososphaerales* (yellow), and *Ca.* Nitrosocaldales (red) based on concatenated sequences of 71 single-copy core genes. The culture genomes and MAGs obtained in this study are highlighted in bold. Confidence values are on the basis of 100 bootstrap replications. The scale bar represents 10% estimated sequence divergence. Total gene numbers are displayed as histogram plots for each AOA species genome with core genes shown in red and unique genes shown in blue.

A total of 44 genomes sequenced from AOA cultured species or extracted from environmental metagenomes, representing all known ammonia-oxidizing thaumarchaeotal orders, were analyzed and compared in the present study (Table S1). In addition to the 30 genomes that have been previously reported, seven marine and two terrestrial AOA culture genomes as well as five MAGs from marine, geothermal, and engineered systems were obtained in this study (Table S1). These new genomes originate from diverse geographic locations and habitats, including fresh and coastal marine water columns and sediments, deep ocean trench waters, and hot springs (Fig. S1). To infer the most probable evolutionary relationship among AOA strains and refine the taxonomy of the unresolved thaumarchaeotal taxa, we constructed a maximum likelihood phylogenomic tree using a multiple sequence alignment of 71 single-copy core genes shared among all 44 AOA genomes (Fig. 1 and Table S2). While the topology of this species tree was broadly congruent with those of the single-gene trees based on 16S rRNA and *amoA* genes, forming four basal order-level lineages within the phylum *Thaumarchaeota* [26, 27, 36, 37], the higher resolution of the phylogenomic analysis clearly differentiated closely related *Nitrosopumilus* strains (Fig. 1). The same speciation phylogeny of AOA was evident from the phylogenomic tree including 21 additional high-quality (completeness >90% and contamination <5%) AOA MAGs and SAGs [38–40] (Fig. S2).

The phylogenetic position of a long-branching taxon, *Ca*. Cenarchaeum, has remained largely ambiguous, because the extensive gene exchange between the only representative of the genus, *Ca*. Cenarchaeum symbiosum [8], and its sponge host confounds the inference of the evolutionary history of the lineage. However, the availability of a free-living *Ca*. Cenarchaeum strain, HMK20, has better defined the phylogenetic affiliation of this lineage. The *Ca*. Cenarchaeum genus appears to have diverged from the same lineage as the *Nitrosopumilus*/*Nitrosarchaeum* genera (Figs. 1 and S2). Similarly, the uncertain affiliation of *Ca*. Nitrosomarinus catalina SPOT01, a novel marine AOA strain enriched from California coastal waters [41], has been resolved. Although earlier proposed to represent the unique *Ca.* genus Nitrosomarinus, our phylogenomic analysis placed it well within the genus *Nitrosopumilus* (Figs. 1 and S2), and the average nucleotide identity (ANI) values between *Ca*. Nitrosomarinus and *Nitrosopumilus* genera (75–79%) were comparable to many ANI values within *Nitrosopumilus* genus (76–79%) (Fig. S3 and Table S3), suggesting that these two genera are phylogenetically indistinguishable. Given the genus *Nitrosopumilus* was described first [26], we suggest that the *Ca.* genus Nitrosomarinus is a later heterotypic synonym of the genus *Nitrosopumilus*. In addition, two previously defined distinct thermophilic AOA species, *Ca*. Nitrosocaldus islandicus 3F and *Ca*. Nitrosocaldus cavascurensis SCU2 [42, 43], share more than 99.8% similarity across their genomes (Fig. S3 and Table S3). Thus, this pair of genomes only sample subspecies level diversity, and the denomination should be refined. An unexpected finding was that the MAGs recovered from the hadopelagic waters (>6000 m) of the oceanic trenches (YT1, F8–1, and F8–2) were closely grouped with *Nitrosopumilus* genus and widely separated from other oceanic AOA taxa that comprise *Ca*. Nitrosopelagicus strains and uncultured water column B (WCB)-AOA populations (O23) (Figs. 1 and S2).

Comparative analysis revealed extensive gene content and genome size variation among marine and terrestrial AOA genomes. For example, the gene content and genome sizes of *Ca*. Nitrosocosmicus-like soil AOA species (3395–3758 genes; 2.99–3.43 Mbp) are nearly threefold greater than those of *Ca*. Nitrosopelagicus (WCA) and WCB-like oceanic AOA species (1400–1502 genes; 1.17–1.25 Mbp) (Fig. S4 and Table S1). In addition to their distinct genome sizes, the genome coding density of AOA species varied widely, ranging from 73.7 to 93.9% (Fig. S4). The genome coding density of all AOA species together showed a clear linear decrease with increasing genome size (Fig. S4). Consistent with other oligotrophic marine bacterial genomes, such as SAR11, *Prochlorococcus*, and SUP05 (Table S1), the genomes of oceanic AOA species are highly streamlined and relatively gene dense (Fig. S4). In contrast, neutral pH soil AOA species that occupy nutrient-enriched environments tend to have larger genomes, and their genome coding densities are even lower than those of the eutrophic ammonia-oxidizing bacteria species (Fig. S4 and Table S1). The small marine AOA genomes are also associated with generally lower GC content than soil AOA (Fig. S3 and Table S1). Together, these findings indicate that nutrient levels and habitat types both have profound effects on the gene content and organization of AOA genomes.

### The core and pan-genome of the ammonia-oxidizing *Thaumarchaeota*

**Fig. 2:**
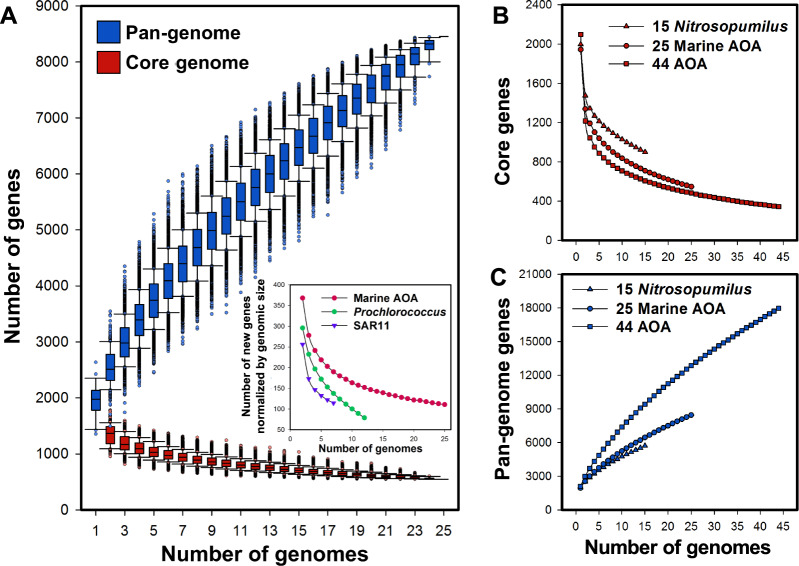
AOA core genome and pan-genome analyses. **a** Box-and-whisker plots of core and pan-genome sizes of 25 marine AOA species. The number of genes is plotted as a function of the number of *n* strains sequentially added. For *n* genomes selected out of 25, there are 25!/[(*n* − 1)!·(25 − *n*)!] possible combinations from which to calculate core and pan-genomes. Each possible combination is plotted as a red and a blue dot for core genome and pan-genome analysis, respectively. To compare the pan-genome openness of marine AOA species with those of *Prochlorococcus* and SAR11 species, the average values of the number of new unique genes per Mb genome was calculated (inset panel) with the sequential addition of each marine AOA (red), *Prochlorococcus* (green), or SAR11 (purple) genomes. In addition to 25 marine AOA species, the average values of the number of core genes (**b**) and pan-genome genes (**c**) are shown for 15 *Nitrosopumilus* species (triangle) and all 44 members of AOA (square), respectively.

To provide further quantitative insights into the conserved and flexible gene pools within the ammonia-oxidizing *Thaumarchaeota*, we calculated the core genome that is shared by all AOA species and the pan-genome that represents the global gene repertoire of AOA (Fig. 2). The core genome of ammonia-oxidizing *Thaumarchaeota* comprises ~344 genes (Fig. 2b), including key pathway genes that are involved in the characterized central metabolism of AOA, such as carbon fixation through the 3-hydroxypropionate/4-hydroxybutyrate cycle and cobalamin biosynthesis [19, 23, 24, 38] (Figs. S5 and S6; Table S4). These core gene sets only represent a small fraction of the genomes of AOA species, accounting for ~14–23% of the genes in *Nitrosopumilales* genomes (62–99% ANI), ~9–12% in *Nitrososphaerales* genomes (64–86% ANI), ~14–19% in *Ca*. Nitrosotaleales genomes (78–83% ANI), and ~19–22% in *Ca*. Nitrosocaldales genomes (78% ANI). These low core gene set fractions suggest a large proportion of AOA genomes could be associated with environmental-specific functions that may provide fitness advantages in distinct marine and terrestrial habitats.

The ammonia-oxidizing thaumarchaeotal pan-genome within the available dataset contains a total of ~17,961 genes, and the comparative analysis of 44 AOA species genomes revealed that sampling of their pan-genome is far from saturated (Fig. 2c). The AOA pan-genome possesses a high proportion of putative and hypothetical genes with unknown functions (Fig. S7). The accessory and unique genes that are assigned to the Clusters of Orthologous Groups (COGs) functional categories amino acid transport and metabolism, transcription, and energy production and conversion are among the most abundant genes that contribute to the global pool of AOA genes (Fig. S7). When considering only the 25 marine AOA species genomes with an ANI range of ~62–99%, the pan-genome consists of nearly 8500 genes (Fig. 2a). An average of ~111 novel unique genes per Mb are expected to be identified with each new marine AOA strain sequenced (Fig. 2a). Open pan-genomes with large genetic repertoires have also been observed for the other two most abundant marine microbial groups, SAR11 and *Prochlorococcus* [44, 45]. Notably, the number of new genes per Mb marine AOA species genome is even greater than those calculated for SAR11 and *Prochlorococcus* species, although they have a comparable degree of genome divergence (~64–97% of ANI) (Fig. 2a and Table S3). These findings further highlight the extensive genomic diversity among the globally abundant archaeal ammonia oxidizers.

### Functional analysis of core and flexible genes in ammonia oxidation and assimilation pathways

**Fig. 3:**
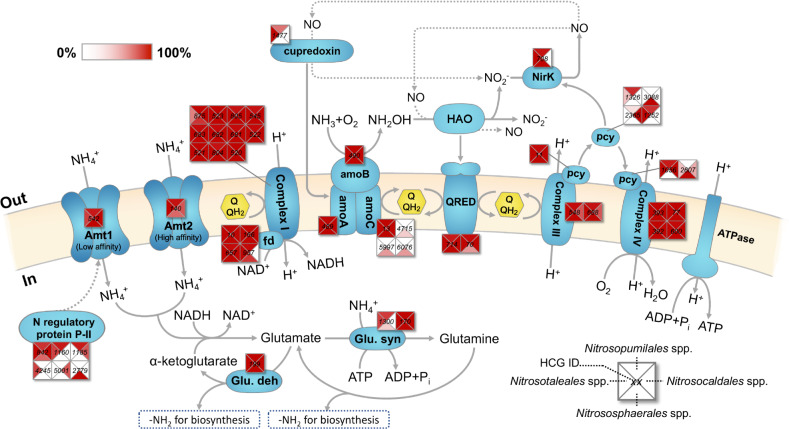
Reconstruction of the proposed pathways of ammonia oxidation, electron transfer, and ammonia assimilation in AOA emphasizing the conservation and uniqueness of pathway enzymes of AOA species. Alternative archaeal ammonia oxidation models are illustrated based on the current literature on pathway gene identification [29], identification of pathway intermediates [16, 56, 79], isotopic measurements [21, 56], and transcriptional regulation [28, 52]. Squares indicate the COGs of proteins in AOA species genomes, and COG numbers are inside squares. Each square is split into four pieces to represent AOA species affiliated to the orders *Nitrosopumilales*, *Nitrososphaerales*, *Ca*. Nitrosotaleales, and *Ca*. Nitrosocaldales. Color scale reflects the percent of genomes in each ammonia-oxidizing thaumarchaeotal order with that COG. QRED quinone reductase, HAO putative hydroxylamine oxidoreductase, pcy plastocyanin, fd ferredoxins, Amt1 and Amt2 low-affinity and high-affinity ammonia transporter, respectively, Glu. deh and Glu. syn glutamate dehydrogenase and glutamine synthetase, respectively.

Despite their extraordinary genomic divergence, all AOA species share a common pathway for energy generation by oxidation of ammonia. We found that many essential components of ammonia oxidation, electron transfer, and ammonia assimilation pathways are mostly conserved across all marine and terrestrial AOA genomes, including A and B subunits of ammonia monooxygenase (AMO), quinone reductase, the conventional complex III and IV, ferredoxin and complex I (NADH:ubiquinone oxidoreductase), glutamine synthetase, and glutamate dehydrogenase (Fig. 3 and Table S4). In contrast, other genes inferred to have functions in regulation, stress responses, and ammonia uptake are highly variable among different orders of AOA species (Fig. 3 and Table S4). For instance, only *Nitrososphaerales* species possess extra copies of AmoC homologs hypothesized to provide a chaperone-like function to maintain the structural integrity of AMO holoenzyme under nutrient-depleted conditions [28] (Fig. 3). Given the generally low affinity of *Nitrososphaerales* species for ammonia [46], they may frequently face energy limitation or starvation. Thus, this adaptive genomic feature may allow *Nitrososphaerales*-like AOA to cope with intermittent energy stress in soils. Within the order *Nitrososphaerales*, *Ca*. Nitrosocosmicus species were isolated from the high nutrient environments of fertilized soils and wastewater treatment systems [47–49]. Intriguingly, *Ca*. Nitrosocosmicus species lack the high-affinity ammonium transporter and S-layer proteins associated with the high ammonia affinity of marine AOA species (Table S4) [28, 32, 50]. The absence of these genes suggests they are adapted to much higher ammonia concentrations than is typical of most natural environments.

Since ammonia is used both as an energy source in ammonia oxidation and a nitrogen source in biosynthesis, sophisticated regulation of these two processes must maintain the metabolic and anabolic balance in AOA cells during adaptation to different redox and nutrient conditions. We found all AOA species encode an extensive gene repertoire for plastocyanin-like proteins that appear integral to respiration and electron transfer reactions associated with ammonia oxidation, as well as PII proteins that are predicted to be involved in regulation of ammonia assimilation (Fig. 3 and Table S4). High expression of many of these genes has been observed in both transcriptomes and proteomes of marine and terrestrial AOA species [28, 34, 51–54], suggesting they serve essential roles in respiratory and biosynthetic activities in AOA. We found large variations in the gene sets encoding plastocyanin proteins and PII proteins among different orders of AOA species (Fig. 3 and Table S4). Thus, these proteins are potentially under strong diversifying selection throughout the evolution of *Thaumarchaeota*, and genomic variation in systems of ammonia oxidation, electron transfer, and nitrogen assimilation likely reflect ecophysiological differences that determine habitat preference.

Nitric oxide (NO) is a central intermediate in the archaeal ammonia oxidation pathway, and the interaction between NO and cobalamin plays an important role in shaping the general ecophysiology of AOA species under stressed conditions [16, 55, 56]. One candidate enzyme for the production of NO in AOA is the putative copper-containing nitrite reductase (NirK). NirK genes are highly expressed during exponential growth of marine and soil AOA cells [28, 34, 51, 52], and the expression of *nirK* genes is tightly regulated by ammonia availability, suggesting NirK serves a key role in AOA ammonia catabolism [28]. However, although *nirK* genes are widely distributed in marine and soil AOA, no NirK homolog has yet been identified in any *Ca*. Nitrosocaldales species from geothermal systems (Table S4) [42, 43]. Since the ammonia oxidation by *Ca*. Nitrosocaldales species is highly sensitive to low concentrations of the NO-scavenging chemical PTIO (2-phenyl-4,4,5,5,-tetramethylimidazoline-3-oxide-1-oxyl) [42, 43], NO appears to be an obligate intermediate or reactant in ammonia oxidation by all AOA lineages. Thus, the role of NirK in the pathway of archaeal ammonia oxidation is unresolved.

Apart from the conservation of *nirK* homologs in all known mesophilic AOA, its functional importance was also indicated by the emergence of a *nirK* variant containing a nonsynonymous mutation after extended laboratory cultivation of *N. maritimus*. Since the isolation of *N. maritimus* strain SCM1 in 2005, this strain has been continuously transferred under optimum growth conditions over 11 years, representing more than 3000 generations (Fig. S8) [17, 25, 26, 57]. Related to extended laboratory passage, the average generation time of SCM1 culture has decreased from ~2 days in 2007 to ~1 day in 2011 and stabilized at around 1 day by 2018 (Fig. S8). To identify mutations associated with the increased growth rate, we compared the genome sequence of the ancestral SCM1 cultures collected in 2007 with those of the evolved cultures collected in 2011 and 2016 after ~1200 and ~3000 generations of evolution, respectively (Fig. S8). In total, we observed 11 genes that harbored nonsynonymous mutations and seven genes with synonymous mutations across ~3000 generations (Table S5). This leads to a mutation rate estimate of marine AOA in the laboratory of 4.3 × 10^–9^ per site per generation, which is higher than that reported for *Prochlorococcus* (~10^−10^ per site per generation) and at the higher end of previous estimates for other bacteria (0.8 × 10^−10^–9.8 × 10^−9^ per site per generation for 26 species) [58, 59]. Three of the 11 genes with nonsynonymous mutations are in the ammonia oxidation and assimilation pathways, including genes encoding putative NirK (Nmar_1667), a low-affinity ammonia transporter (AMT) (Amt1; Nmar_0588) that mutated within ~1200 generations, and glutamate dehydrogenase (GDH) gene that mutated sometime during ~1800 additional generations of laboratory cultivation (Table S5). All mutations in genes for ammonia oxidation and assimilation are close to fixation, present at >96% frequency, and therefore likely conferred a fitness advantage to *N. maritimus* growing under laboratory culture conditions of relatively high ammonia concentration (Table S5).

Several observations are consistent with a significant functional impact of the mutation. The glycine to serine mutation we observed in NirK is near the copper-binding site of this enzyme (Table S5), and microsensor measurements of NO concentrations showed a higher initial accumulation rate of NO in cultures with the NirK mutation than earlier observed in cultures of lower laboratory passage (Fig. S9). This is suggestive of a direct involvement of NirK in NO metabolism. If the current model for archaeal ammonia oxidation is correct [28, 56, 60], a higher rate of enzymatic NO production might alleviate a kinetic limitation imposed by NO availability in the oxidation of ammonia to hydroxylamine, leading to the significantly higher specific growth rates that we observed in evolved cultures (Fig. S8). Similarly, the mutation in the AMT could reflect altered function associated with continuous growth under relatively high concentrations of ammonia not typical of environmental availability.

### Global distribution of the major genotypes of marine AOA

**Fig. 4:**
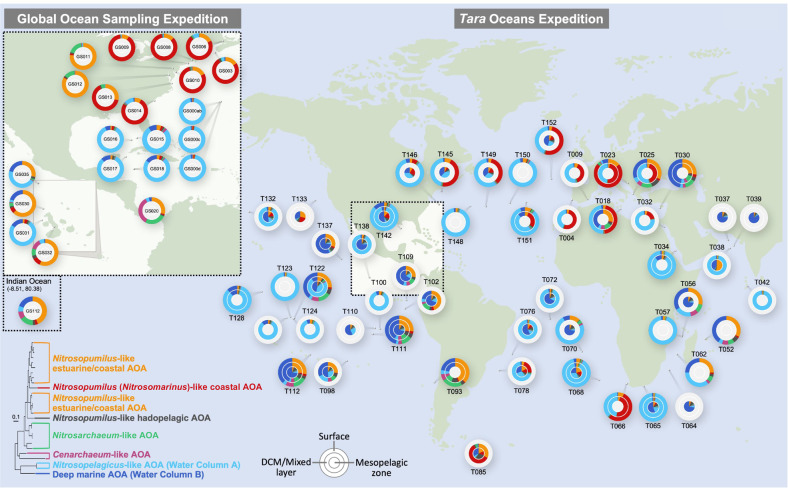
The global distribution of major marine AOA genotypic groups across the upper oceans. Twenty-five marine AOA species were clustered in seven separate genotypic groups based on their positions in the phylogenomic tree and their distinct geographic origins (Fig. S10). *Tara* Oceans and GOS (inset panel) metagenomic reads were recruited to a genomic database containing seven marine AOA genotypic groups to assess the distribution of marine AOA genotypes spanning multiple depth layers of the upper ocean. Only the top hits with a maximum *E*-value of 1e^−^^10^ and a minimum amino acid identity to reference species genomes of 80% were retained for competitive fragment recruitment analysis. The fraction of metagenomic reads recruited to marine AOA genotypes from the surface water layer, deep chlorophyll maximum (DCM)/mixed layer, and mesopelagic zone are depicted on the outermost ring, middle ring, and inner ring at each GOS and *Tara* Oceans sampling station, respectively. The concentric rings are colored as light gray for the station depth layers without metagenomic data collection.

The comparative analysis of 25 marine AOA species genomes allowed us to define seven major genotypic groups (potential functional guilds) of marine AOA that represent the dominant populations in estuarine and coastal areas, surface and deep open oceans, and hadopelagic waters at depths below 6000 m (Fig. S10). In order to analyze the distribution patterns of these genotypes spanning the global upper oceans, we used competitive fragment recruitment to estimate the relative recruitment to the genomes of marine AOA genotypes in global ocean metagenome datasets, including the Global Ocean Sampling (GOS) expedition data and *Tara* Oceans Global expedition data (2009–2013). The GOS expedition contains metagenome datasets recovered from surface water samples, and we found that the dominant marine AOA population shifted along the inshore–offshore gradient (Fig. 4). *Nitrosopumilus*-like AOA were most abundant in estuarine areas, whereas they were of lower abundance in further offshore waters (Fig. 4). *Nitrosopumilus* (*Ca*. Nitrosomarinus)-like AOA were the dominant genotype in coastal areas, while *Ca*. Nitrosopelagicus-like AOA represented the major population in open ocean surface waters (Fig. 4). *Tara* Oceans expedition conducted a more extensive global scale metagenome survey at multiple depths across diverse oceanic provinces. The overall distribution and diversity patterns of the marine AOA communities of *Tara* Oceans’ surface water samples were broadly similar to those of GOS samples (Fig. 4). Apart from metagenome data collected from shallow waters, the *Tara* Oceans survey extended to mesopelagic depths (250–1000 m), where the WCB genotype was found to dominate marine AOA communities (Fig. 4). It is worth noting that, as has been reported in Monterey Bay surface waters [61], the WCB genotype appeared to be abundant at shallow depths in many upwelling regions (Fig. 4).

**Fig. 5:**
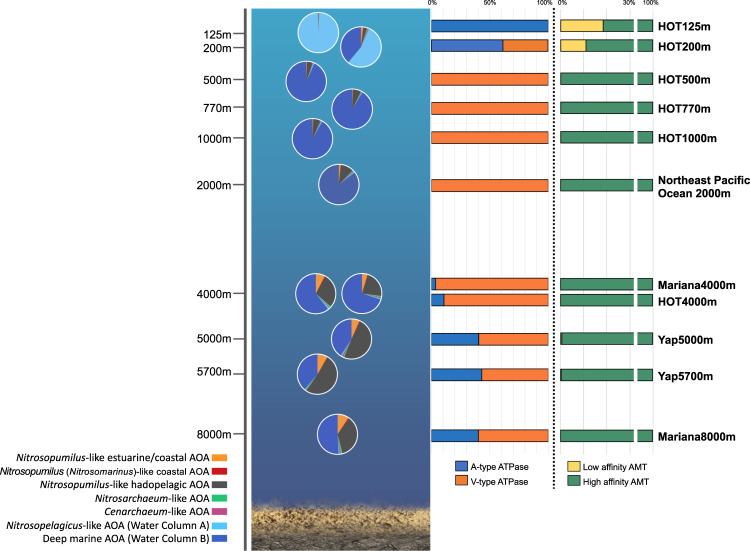
Depth distribution of marine AOA genotypic groups (pie chart) as well as associated ATPase and ammonia transporter (AMT) types (bar chart) from epipelagic to hadopelagic waters. Metagenomic reads from the North Pacific HOT time-series station (125–4000 m; HOT cruise 229), Northeast Pacific Ocean (2000 m), the Yap Trench of the western Pacific (5000–5700 m), and the Mariana Trench waters (4000–8000 m) were recruited to a genomic database of major marine AOA genotypic groups to assess the distribution of marine AOA genotypes as well as associated ATPase and AMT types along the whole water column. Genotype designations and the corresponding color schemes are the same as those shown in Fig. 4.

In addition to investigating the global surface distribution of marine AOA genotypic groups in the upper oceans, we analyzed the vertical distribution of these lineages from the epipelagic zone of station ALOHA to the hadopelagic waters of Mariana Trench and Yap Trench at depths down to 8000 m (Fig. 5). In the well-stratified water column of station ALOHA, we found a major population shift within a narrow depth interval from 125 m, where *Ca*. Nitrosopelagicus-like AOA dominated to 500 m, where WCB genotype dominated (Fig. 5). WCB genotype was consistently abundant throughout the dark ocean water column (Fig. 5). In the deep trench waters, a lineage that is closely related to *Nitrosopumilus* genus appeared to be one of the most abundant AOA genotypes (Fig. 5). This genotype was not solely restricted to the hadal zone, and they also constitute a substantial proportion of the total AOA population in energy-depleted bathyal and abyssal zones (Fig. 5). This hadopelagic genotype was well separated from other pelagic AOA lineages (*Ca*. Nitrosopelagicus-like AOA and WCB), but shared more than 98% 16S rRNA gene sequence identity with estuarine and coastal *Nitrosopumilus* species and formed a well-supported monophyletic sister group to the *Nitrosopumilus* genus in the phylogenomic tree (Figs. 1 and S2). Thus, *Nitrosopumilus*-like AOA appear to occupy a wide range of marine habitats and depths that correspond to distinct nutrient regimes, temperatures, and pressures. We hypothesize that genetic adaptations are responsible for their substantial expansion into distinct ecological niches.

### Genetic diversification associated with niche adaptation in marine AOA

To identify accessory and unique gene contents that are associated with the habitat-specific adaptations as well as abiotic and biotic selective forces that shape genomic heterogeneity among marine AOA populations, we linked the geographic distribution of marine AOA genotypic groups with patterns of variable gene content involved in stress response (Fig. S11 and Supplementary information), nutrient uptake, and metabolic flexibility (Fig. S12 and Supplementary information). A recent comparative analysis of ATPase gene clusters revealed that different marine AOA genotypes contain two distinct types of ATPase with significant variation in subunit composition and structure [62]. *Ca*. Nitrosopelagicus-like AOA encode archaeal-type (A-type) ATPase, while WCB-AOA from the deep ocean possess vacuolar-like (V-type) ATPase [62]. Although the conserved A-type ATPases were found in both closely related *Nitrosopumilus*-like estuarine/coastal AOA and hadopelagic AOA genotypes, an additional V-type ATPase is exclusively present in hadopelagic AOA, suggesting that V-ATPase in *Nitrosopumilus*-like AOA may play a key role in their adaptive expansion to the deep ocean [62].

Consistent with cultured AOA species, environmental marine AOA populations contain two ATPase variants that fall into distinct phylogenetic clusters (Fig. S13A). We examined the distribution of marine AOA A-type and V-type ATPase genes across metagenome datasets from epipelagic to hadopelagic waters. We found that the vertical distribution pattern of these two distinct types of ATPase genes broadly matches those of the marine AOA genotypes along the water column (Fig. 5). There was an apparent shift in ATPase composition from A-type in the epipelagic zone to V-type in the upper mesopelagic zone (Fig. 5). V-type ATPase predominated between the mesopelagic and bathypelagic zones (500–4000 m) (Fig. 5). Because *Nitrosopumilus*-like hadopelagic AOA species contain both types of ATPase, as the relative abundance of this genotype gradually increased from the bathypelagic to hadopelagic zone, the fraction of A-type ATPase increased, reaching up to 40% of the total ATPase gene reads in metagenomic samples below 5000 m (Fig. 5). Therefore, we extended the original inference of ATPase occurrence in selected marine AOA species, providing a more complete view of the vertical distribution of ATPase gene clusters across metagenomes. The depth partitioning of two distinct types of ATPase revealed by our analysis further supports the ecological role of V-ATPase in adaptation to environmental conditions specific to the deep oceans. Wang et al. (2019) suggest that the acquisition of V-type ATPase in deep marine AOA via horizontal operon transfer confers an adaptive advantage in the deep ocean with elevated hydrostatic pressure, as the proposed function of V-ATPase in pumping excessive cytoplasmic protons at high pressure may serve to maintain the cytosolic pH homeostasis in marine AOA.

Another notable finding of the depth distribution of marine AOA genes is the absence of genes encoding the low-affinity (high *K*_m_) AMT in the deep ocean. Although both low- and high-affinity *amt* genes are present in all cultivated marine AOA species [50, 63], the high-quality MAGs recovered from mesopelagic (O23) and hadopelagic waters (YT1, F8–1, and F8–2) lack low-affinity *amt* genes (Table S4). Our metagenomics survey of the entire water column further revealed that low-affinity AOA *amt* genes were restricted to the epipelagic zone and rarely detected at depths below 200 m; the high-affinity AMT was the only type of ammonia uptake system in marine AOA populations inhabiting the ammonia-depleted deep oceans (Fig. 5). In our previous works, we showed that *N. maritimus* sustained high expression levels of the high-affinity *amt* gene at low nanomolar ammonia concentrations or under short-term ammonia starvation, whereas the expression of low-affinity *amt* was depressed under these conditions, indicating that *N. maritimus*, although possessing both types of ammonia uptake systems, selectively retains high-affinity AMT in response to extremely low or even no ammonia supply [28, 50]. Furthermore, our recent findings indicated that the in situ affinity of marine AOA for ammonia increased with depth overall [64]. Consistently, high-affinity AMT would confer a stronger selective advantage over low-affinity AMT for WCB-AOA and *Nitrosopumilus*-like hadopelagic AOA in the deep oceans, supporting their exceptionally high substrate affinities (low *K*_m_ values) at depth where ammonia concentrations and fluxes are extremely low or even undetectable [64].

We also found that the phylogeny of high-affinity *amt* genes of marine AOA strains tracks habitat, not organismal phylogeny inferred from conserved single-copy genes. The high-affinity *amt* genes of *Nitrosopumilus*-like hadopelagic AOA were not clustered with those encoded by estuarine/coastal *Nitrosopumilus* species, but rather formed a monophyletic group with those of pelagic AOA (Fig. S14). Thus, in addition to V-ATPase, the acquisition of high-affinity AMT from pelagic AOA may also play a significant role in the adaptive expansion of closely related *Nitrosopumilus*-like AOA populations from coastal waters to the oceanic trenches.

The phosphorus utilization capacity in AOA has been poorly characterized relative to their nitrogen utilization and carbon fixation capacities. Many AOA species possess two sets of scavenging systems for orthophosphate, the high-affinity *pst* transporter (*pstABCS*), which is regulated by *phoU*, and low-affinity *pit* transporter, which is regulated by *pit* accessory proteins (Fig. S15). Although a putative phosphonate transporter gene cluster (*phnCDE*) was found in AOA genomes, the lack of an identifiable C-P lyase gene and other described phosphonate hydrolase genes suggests that *phnCDE* encode a transport system for a different substrate in AOA or may be nonfunctional (Fig. S15). The presence of genes encoding polyphosphate utilization (*ppA* and *ppX*) may be responsible for using an intracellular P-reserve under P-limited conditions (Fig. S15).

Although phosphorus concentrations are high in the energy-limited deep oceans, phosphorus can be extremely limited in the upper water column of many oceanic regions [65–67]. Phosphorus deficiency has been increasingly recognized as an important factor that controls community structure and drives genome differentiation of marine microorganisms [68–70]. We used *Tara* Oceans metagenomes to investigate the influence of phosphorus availability on the oceanic distribution of P-acquisition gene content in marine AOA. Notably, the recruitment analysis of AOA P-acquisition genes versus *amoA* genes showed that the frequencies of high-affinity (low *K*_m_) and low-affinity (high *K*_m_) phosphate transporter genes were strongly associated with phosphate concentrations in the upper ocean. High frequencies of the AOA *pstB* gene that encodes the ATP binding subunit of the high-affinity P transporter were enriched in *Tara* Oceans samples with low phosphate concentrations of less than 200 nM (0.54 ± 0.26 estimated copy number per cell) (Fig. S16). However, significantly lower frequencies (*p* < 0.01) of AOA *pstB* genes were found in high phosphate samples (0.2–3.3 μM) (Fig. S16). In contrast, the low-affinity P transport system (*pit* genes) appears to be part of the core genetic ensemble of marine AOA populations in high phosphate regions, with an average estimated frequency of ~0.83 copy number per cell; while frequencies of AOA *pit* genes were significantly lower in samples with low phosphate concentrations (<200 nM) relative to high phosphate (*p* < 0.01) (Fig. S16).

**Fig. 6:**
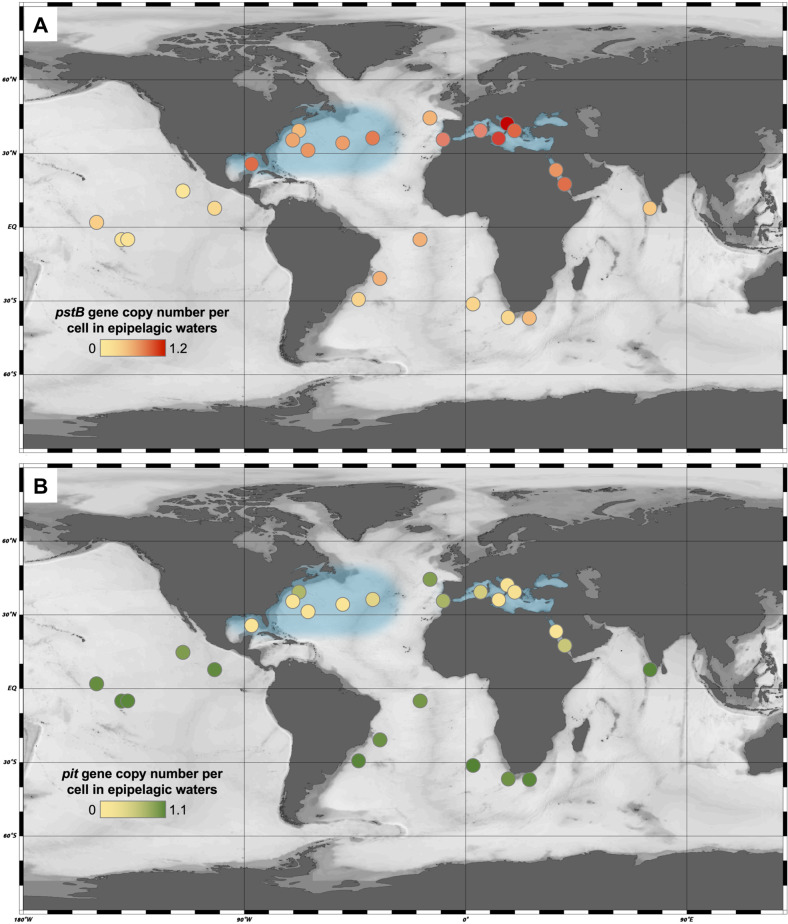
The relative enrichment of high-affinity *pstB* genes and low-affinity *pit* genes in marine AOA populations from the upper water column of diverse oceanic regions. The per-cell gene abundance was estimated based on the relative ratios of *pstB* (**a**) and *pit* (**b**) to *amoA* genes recovered from each *Tara* Oceans sampling station by metagenomic read recruitment, assuming 1 *amoA* gene copy per marine AOA genome. AOA *pstB* gene was enriched in the surface and DCM waters within the low phosphorus regions highlighted by shading, such as the western North Atlantic and the Mediterranean Sea.

No gene encoding the high-affinity P transport system was found in the streamlined genomes of *Ca*. Nitrosopelagicus brevis strains CN25 and U25 recovered from shallow waters of the North Eastern Pacific (Table S4). AOA *pstB* genes were only enriched in upper waters of the oceanic regions with extremely low phosphate concentrations, such as the western North Atlantic and the Mediterranean Sea [65, 66], where marine AOA are expected to constantly experience intense competition for limited phosphate (Fig. 6a). Thus, marine AOA in these oceanic regions appear better equipped to cope with P-limitation than those in high-phosphate regions. Most of the AOA *pstB* genes recovered from these P-depleted oceanic regions formed a monophyletic group that is distinct from those of the cultured AOA species (Fig. S13B). In addition, the frequency of AOA *pit* genes was found to be the lowest in these low phosphate regions (Fig. 6b). The loss of low-affinity P transporter genes is likely a consequence of the transporter having no or limited selective advantage in highly oligotrophic environments [71]. Together, our findings indicate that P availability is a dominant selective force that drives genomic diversification among marine AOA natural populations and revealed the previously underappreciated role of phosphorus in structuring their distribution and ecology.

It has been shown that *Nitrosopumilus maritimus* is capable of synthesizing methylphosphonic acid (MPn), implicated as a major source of methane in the upper oceans [22]. In addition to *N. maritimus*, we found that five estuarine and coastal marine AOA species (strains HMK29, NM25, SPOT01, BD31, and SFB1) and one freshwater AOA species (DW1) possess the complete or nearly complete MPn biosynthetic pathway (Table S4). Methylphosphonic acid synthase (MPnS) represents the key enzyme to synthesize MPn in AOA (Fig. S17). We found thaumarchaeotal *mpnS* genes are widespread in diverse oceanic provinces and enriched in marine AOA populations in deep water habitats relative to shallow water habitats (Fig. S17A, B). Phylogenetic placement of all identified thaumarchaeotal *mpnS* genes and partial fragments revealed that, distinct from the *mpnS* genes encoded in coastal and terrestrial AOA species genomes, most deep marine *mpnS* gene sequences clustered together as new lineages that are likely associated with the uncultured WCB-AOA populations (Fig. S17C). High abundances of *Sulfitobacter* and *Oleiphilus* species that encode C-P lyase for MPn degradation has been found in the deep-water column at Station ALOHA [72], suggesting that phosphonate cycling may be a significant process in the deep ocean. Our findings suggest that MPn is not only a likely source of methane in the upper oceans, but also may be an important source of methane to deep waters. The produced methane may fuel methane-oxidizing microorganisms in the deep oceans, contributing to the reported decrease in methane concentration and ^13^C enrichment of the residual methane at depth [73].

## Conclusions

By bringing together studies of biogeography, cultivation, laboratory evolution, genomics, and physiology, we developed a more holistic understanding of traits associated with the adaptive radiation of AOA into a wide variety of habitats. In particular, by linking the environmental distribution of the major genotypes of marine AOA with their variable gene contents, we identified several variable genes that determine traits associated with ecosystem-specific selection pressures in different oceanic regions. In addition to the previously recognized key environmental variables that control the distribution and diversity of marine AOA populations, such as ammonia concentrations [18, 74], light levels [51, 75], and reactive oxygen species [75–78], our data indicate that phosphate concentrations and hydrostatic pressures drive marine AOA genotypic and gene content variation in the ocean. Likewise, P availability has been identified as a major ecosystem-specific selective pressure that shapes the P-related gene content and gene sequences of another two most abundant marine microbes, *Prochlorococcus* and SAR11 [69]. Our findings reinforce the importance of the acquisition of beneficial nutrient scavenging genes as a common adaptive strategy for marine oligotrophs in nutrient-limited regions of the ocean. Unlike the primary association of *Procholorococcus* and SAR11 with temperate aquatic environments, AOA are widely distributed—from thermophilic to mesophilic habitats and from terrestrial to marine systems. Our results show that extensive horizontal transfer of genes and entire operons is closely associated with their habitat expansion, likely facilitating their adaptive radiation into a variety of ecological niches, including those spanning a range of temperature, pH, pressure, and nutrient availability.

It has been suggested that AOA play a significant role in shaping biodiversity in marine environments by controlling the forms of fixed nitrogen species available to other microbial assemblages and supplying vitamins to vitamin-dependent populations in the ocean [16, 23]. Our data suggest that interactions between the co-occurring WCB-AOA and bacteria encoding C-P lyase may be important to phosphorus cycling and as a source of methane in the deep ocean. Our comparative genomics and metagenomics analyses should also guide future isolation studies, suggesting that new cultivation strategies, such as high-pressure selection, are possibly required to culture piezotolerant or piezophilic species. In turn, the culture collection of environmental representatives of marine AOA and associated biota will serve to establish the model systems to investigate how mutualistic or competitive interactions between these dominant taxa and other organisms influence the biogeochemistry of marine and terrestrial ecosystems.

## Supplementary information

Supplementary Text

Supplementary Figures

Table S1

Table S2

Table S3

Table S4

Table S5

Table S6
