# Supplementary material for: Alternative strategies of nutrient acquisition and energy conservation map to the biogeography of marine ammonia-oxidizing archaea
Source: ISME J. 2020 Jul 7;14(10):2595–609. doi: 10.1038/s41396-020-0710-7 (PMC7490402; doi:10.1038/s41396-020-0710-7)
Supplement: Supplementary file 1 — Supplementary Text [file 41396_2020_710_MOESM1_ESM.pdf]

Supplementary Information

Results and Discussion

A notable feature of *Nitrosopumilus* physiology is the demonstrated ability of *Nitrosopumilus maritimus* to synthesize ectoine (1,4,5,6-tetrahydro-2-methyl-4-pyrimidinecarboxylic acid) and hydroxyectoine in response to osmostress [1]. Our comparative genomics indicated that the complete biosynthetic gene cluster (*hyp-ectABCD*) was not restricted to the genus *Nitrosopumilus*, but also occurred in some species from the genera *Nitrosarchaeum* and *Ca*. Cenarchaeum (Table S4). Previous metagenomics studies reported that ectoine/hydroxyectoine biosynthetic gene clusters were present in halotolerant marine AOA populations from the brine-seawater interface [2]. Our broad metagenomics survey further revealed the widespread distribution of the genetic capacity of ectoine/hydroxyectoine biosynthesis in marine AOA populations spanning estuarine, coastal and open ocean surface waters (Fig. S11). Like other osmolytes, ectione likely serves additional functions beyond osmoprotection and these functions may be selected for in different environments. For example, in bacteria [3, 4], ectoine may serve to protect AOA cells against UV radiation or cytotoxin stresses in surface waters.

It has been recognized that many cultured and environmental AOA populations contain the complete *ure* gene cluster (urease, urease accessory proteins, and urea transporters) and can use urea as a source of ammonia for energy production and growth [5-9]. Recent field studies reported that the in situ specific affinities for urea-based ammonia oxidation were comparable to those for in situ ammonia-based ammonia oxidation in the ocean, indicating that besides ammonia, urea appears to play an important role in fueling marine nitrification [10]. The Hawaii Ocean Time-Series (HOT) station metagenome datasets were used to assess the prevalence of

urea utilizing genes in natural marine AOA communities by calculating the ratio of reads that mapped to AOA *ureC* genes (encoding the alpha subunit of urease) with the criterion of E-value $\leq 1 \times e^{-10}$, reads sequence identity ≥ 80%, and reads length ≥ 100 bp divided by reads that mapped to AOA *amoA* genes (Fig S12). We found that *ureC* genes were distributed throughout the upper ocean water column at the HOT station (125–1,000 m) (Fig. S12). The recruitment ratio of AOA *ureC* genes versus *amoA* genes indicates that ~40–100% of marine AOA contain urea utilizing genes (Fig. S12). Similarly, high *ureC*:*amoA* ratios were also observed in the equatorial Pacific Ocean (22–55%) [11], the Gulf of Mexico (~10–15%) [8], and the San Pedro Ocean Time-Series (SPOT) site (~60–100%) [12], together highlighting the pervasiveness and importance of urea as an alternative substrate for AOA in the ocean.

Materials and Methods

Sample source, culture maintenance and genome sequencing

All AOA species were maintained in liquid mineral medium in the dark without shaking. *Nitrosopumilus ureiphilus* strain PS0, *Nitrosopumilus cobalaminigenes* strain HCA1, and *Nitrosopumilus oxyclinae* strain HCE1 were cultured in HEPES buffered synthetic crenarchaeota medium (SCM) supplemented with 0.2–1 mM NH_4Cl and 100 µM α-ketoglutaric acid at 25°C as described previously [13]. Cells were harvested in mid-exponential phase via vacuum filtration on 0.22 µm Sterivex-GP (Millipore Corporation, Billerica, MA, USA) filters for genome DNA extraction. To minimize DNA shearing, the sucrose lysis method was used for DNA extraction. In brief, the filter was removed from the Sterivex cartridge, aseptically cut into small pieces with flame-sterilized scissors, and transferred to a sterile 15 ml Falcon tube. Two milliliters of fresh sucrose lysis buffer containing 50 mM Tris-HCl at pH 8.0, 40 mM EDTA at pH 8.0, and 0.75 M

sucrose was added to the Falcon tube. Immediate flash freezing of the sample in liquid nitrogen and subsequent thawing at room temperature were conducted to release DNA from AOA cells. After one freeze and thaw cycle, 465 µl of 10% SDS and 15 µl of 20mg/ml proteinase K were added, and the contents were mixed by vortexing for 2 minutes. The tube was incubated at 37°C for 2 hours with agitation at 30 minutes intervals. The aqueous phase was transferred to a spin column, and DNA was collected using Qiagen Blood & Tissue kit according to the manufacturer's recommendations.

Genomic DNA was sequenced by a combination of the Illumina MiSeq platform with paired-end (PE) reads of 250 bp and the Pacific Biosciences (Pacbio) RSII SMRT sequencing platform using a 20 kb SMRTbell template library. *De novo* assembly of the Pacbio sequence reads was conducted using the Hierarchical Genome Assembly tool in the PacBio SMRT Analysis Software (RS_HGAP_Assembly.3) [14]. Illumina sequence reads were trimmed and filtered by Trimmomatic (version 0.36) to remove low-quality reads [15]. The initial Pacbio-based assemblies were error corrected with high-quality Illumina reads using Pilon (version 1.23) [16]. The corrected genomes were annotated through NCBI Prokaryotic Genome Annotation Pipeline [17].

Ca. Nitrosarchaeum sp. strain AC2 and *Ca*. Nitrosotenuis sp. strain DW1 were enriched from near-shore sediments of Lake Acton (39°57'N, 84°74'W, USA) and Lake Delaware (40°39'N, 83°05'W, USA), respectively [18]. These two freshwater AOA enrichment cultures were maintained in HEPES buffered freshwater SCM supplemented with 0.5 mM NH_4Cl at 30°C under dark as described previously [18]. Cells were harvested at mid-exponential phase and

genomic DNA was extracted using a sucrose lysis method as described above. DNA extracted from each culture was sequenced on a SMRT cell using PacBio Sequel platform with insert size of approximately 10 kb. The Pacbio sequence reads were clustered according to the Latent Strain Analysis algorithm [19], and the clustered reads were *de novo* assembled by CANU (version 1.8) [20]. The longest contig obtained from each culture was circularized containing an overlapping region of more than 2,000 bp at both ends of the contig. Two complete genomes were annotated through NCBI Prokaryotic Genome Annotation Pipeline [17].

Pure marine AOA culture *Ca.* Nitrosopumilus sp. strain HMK28 was isolated from Bonita beach seawater (26°19'N, 81°50'W) in Estero, Florida. Enrichment cultures *Ca.* Nitrosopumilus sp. strain HMK29 and *Ca.* Cenarchaeum sp. strain HMK20 were obtained from Pine Island seawater (26°37'N, 82°4'W) in Florida and 10 m depth seawater at Lynch Cove (47°37'N, 123°01'W) in Hood Canal, Washington, respectively. These marine AOA cultures were maintained in seawater-based SCM medium supplemented with 1 mM NH_4Cl at 25°C in the dark without shaking. Cells were harvested at late-exponential or early stationary phase, and genomic DNA was extracted using the modified phenol-chloroform extraction method as described previously [21]. Extracted DNA was sequenced using Illumina HiSeq 2000 platform with PE of 150 bp. Illumina sequence reads were assembled using CLC's *de novo* assembly algorithm (version 6.0, CLC Bio, Qiagen, Germany), and the resulting contigs were curated by CodonCode Aligner (version 3.7, CodonCode Corp.) as previously described [22]. Three assembled genomes were annotated with the Rapid Annotation using Subsystem Technology (RAST) pipeline [23], and the annotations were checked and corrected by NCBI Prokaryotic Genome Annotation Pipeline [17].

A pure marine AOA culture of *Ca.* Nitrosopumilus zosterae strain NM25 was obtained from the eelgrass sediments in Tanoura Bay of Shimoda, Shizuoka, Japan [24]. Strain NM25 was maintained in HEPES buffered SCM supplemented with 1 mM NH_4Cl and 100 µM α-ketoglutaric acid [13] at 30°C in the dark with slow stirring. Cells were harvested at late-exponential or early stationary phase via centrifugation, and genomic DNA was extracted from cell pellets with the ISOPLANT II kit (Nippon Gene, Tokyo, Japan) following the manufacturer's instructions. Extracted DNA was sequenced using the Illumina HiSeq1000 platform, and the trimmed reads were assembled using the GS De novo assembler version 2.6 (Roche). The assembled draft genome was annotated with the RAST pipeline [25] and deposited at DDBJ using DDBJ Fast Annotation and Submission Tool (DFAST) [26].

Sampling sites and metagenome-assembled genomes

AOA MAGs ST1, ST2, and ST3 were recovered from activated sludge (AS) samples in the Shatin wastewater treatment plant (Hong Kong, China) that treats saline municipal wastewater. Briefly, three AS samples were collected on October 8th, October 10th, and October 16th, 2013 (ST-1008, ST-1010, and ST-1106). DNA was extracted from these AS samples using FastDNA Spin Kit for Soil (MP Biomedicals, USA) following the manufacturer's instruction and sequenced by Illumina HiSeq 2500 platform with PE of 125 bp and insert size of 300 bp (BGI, China). Quality-filtered (average Q value > 30) metagenome reads of ST-1008, ST-1010, and ST-1016 were co-assembled using CLC's *de novo* assembly algorithm (version 6.04, CLC Bio) with *k*-mer of 35 and contig length > 500 bp. AOA MAGs were recovered using the differential coverage binning method [27]. MAG quality was further improved by using PE tracking to

remove incorrectly binned contigs and recruit the short contigs that were filtered in the preliminary binning process. Scaffolding of contigs in MAGs was performed using SSPACE [15].

Thermophilic AOA MAG US01 was recovered from a hyperthermal hot spring in Ulu Slim, Malaysia. The spring has a near-neutral pH and a maximum temperature at 104°C. Hot spring water and sediment samples were collected in September 2011 at in situ temperature of 90°C. The collected samples were kept at ambient temperature and immediately transferred to the laboratory, where they were stored at 4°C before DNA extraction. Equal volumes of water and sediment samples were used for DNA extraction following the protocol described previously [28]. Extracted DNA was sequenced by Illumina MiSeq with PE of 100 bp and insert size of 300 bp. Quality-filtered (average Q value > 30) metagenome reads were assembled using CLC's *de novo* assembly algorithm (version 6.04, CLC Bio) with *k*-mer of 35 and contig length > 500 bp. A thermophilic AOA MAG was recovered using the differential coverage binning method [27]. Differential coverage of assembled contigs were estimated based on mapping the metagenome reads of sampling site and Sungai Klah hot spring in Malaysia (study accession number: PRJEB7059) to the scaffolds.

Marine AOA MAG YT1 was recovered from 5,000 m water depth in the Yap Trench of the western Pacific (9°52'N, 138°30'E). Briefly, 8 L of seawater was collected by a SeaBird SBE-911 plus CTD in May 2016. Water samples were filtered through a 0.22 µm mesh membrane, and filters were stored at -80°C until further processing. DNA was extracted using a MoBio PowerSoil® DNA Isolation Kit (MO BIO Laboratories, USA) according to the manufacturer's

instructions with a few modifications as previously described [29]. Extracted DNA was sequenced on the Illumina Hiseq X Ten platform with PE of 150 bp. Clean sequence reads were quality trimmed and assembled into contigs using IDBA-UD (Version 1.1.1) with the parameters: -mink 65, -maxk 145, -steps 10 [30]. The ORFs within contigs were predicted by Prodigal with the '-p meta' option (Version 2.6.3) [31]. The initial metagenome binning was performed by MetaBAT (Version 2.12.1) [32] with the modified sensitivity and specificity settings as previously described [33]. A hadopelagic marine AOA MAG was recovered using MetaBAT with the default sensitivity and specificity settings (Version 2.12.1) [32]. Metagenome binning and taxonomic assignments were performed as described elsewhere [33]. Briefly, the initial binning was performed by setting different parameters of sensitivity and specificity in MetaBAT (Version 2.12.1) [32]. Subsequently, all of the bins that retrieved from MetaBAT were pooled for post-dereplication by DAS Tool (Version 1.1) [34].

Hadopelagic marine AOA MAGs F8-1 and F8-2 were recovered from hadopelagic waters in the Challenger Deep region of the Mariana Trench (11°21'N, 142°20'E). In brief, 100 L of seawater for metagenomic analysis was collected from 8,000 m depth by a SeaBird SBE-911 plus CTD in February 2017. Water samples were filtered sequentially through a 3 μm (TSTP, 142 mm, Millipore) and a 0.22 μm (GTTP, 142 mm, Millipore) polycarbonate membrane. Filters were immediately transferred to liquid nitrogen and stored at -80°C until further processing. DNA extracts were obtained with the phenol-chloroform extraction method as previously described [35]. Extracted DNA was sequenced using the Illumina Hiseq X Ten platform with PE of 150 bp and insert size of 350 bp. Clean metagenome reads were quality trimmed and assembled independently using IDBA-UD (Version 1.1.1) with the following parameters: -mink 70, -maxk

100, -steps 10, -pre_correction [30]. Assembled contigs with length > 10 kb were selected for metagenome binning based on the analysis of tetranucleotide frequencies, GC content, and coverage values as described elsewhere [36].

Genomic feature analysis

Completeness, contamination, and coding density of AOA culture genomes and MAGs were assessed by CheckM [37]. Detailed results can be found in Table S1. Average nucleotide identity (ANI) was estimated by pyani.py (https://github.com/widdowquinn/pyani) using BLASTN to align genomic fragments. All predicted genes were searched against the Clusters of Orthologous Group (COG) database [38]. The conserved metabolic pathways shared by ≥ 95% of the complete or nearly complete AOA genomes were constructed based on the Kyoto Encyclopedia of Genes and Genomes (KEGG) reference pathway map [39]. The presence and absence of each pathway enzyme involved in ten major AOA metabolic pathways, such as ammonia oxidation and electron transfer, carbon fixation, phosphorus utilization, and cobalamin biosynthesis, were assessed within available culture genomes and high-quality MAGs (close to or more than 90% completeness and close to or less than 5% contamination) [40]. Detailed results can be found in Table S4.

Core genome and pan-genome analyses

AOA genome proteins were clustered into homolog cluster groups (HCGs) using OrthoMCL based on all-against-all BLASTP with the thresholds of pairwise coverage of 50% and identity of 50% [41]. Orthologs and paralogs were identified as the reciprocal best similarity pairs that were found between and within species, respectively [42]. Core genome represents the HCG genes

shared by all AOA species genomes, MAGs, and SAGs, and pan-genome represents the genes present in at least one AOA genome. To estimate core genome and pan-genome sizes, the shared/unique gene contents with the number of AOA genomes (*N*) ranging from 2 to 45 were determined (N_{max} = 25 for marine AOA species; N_{max} = 12 for *Prochlorococcus* species [43]; N_{max} = 7 for SAR11 species [44]). For each *N*, a total of *C*(45, *N*) combinations of genomes were calculated. If the number of combinations was over 5,000, 5,000 random combinations were sampled for core genome and pan-genome analyses.

Phylogenomic analysis

The phylogenomic trees of AOA were constructed based on concatenated alignments of 71 conserved single-copy homologous proteins from 37 complete or nearly complete AOA cultured species genomes and 7 high-quality AOA MAGs. These marker proteins were identified based on the cluster of HCGs, and the alignments were carried out by MAFFT (version 7.221) [45]. The alignments were edited with Gblocks (version 0.91b) to identify conserved regions [46]. These protein sequences were concatenated as a single evolutionary unit. ProtTest (version 3.4) was employed to select the best-fit model of amino acid substitution according to the AIC and BIC values [47]. Subsequently, the maximum likelihood phylogenomic trees were built by RAxML (version 8.0.26) using the LG+I+G+X model on the basis of 100 bootstrap replications [48].

Evolution experiment and mutation analysis

Since the isolation of *Nitrosopumilus maritimus* strain SCM1 [49], it has been continuously transferred from 2007 to 2018 in HEPES buffered SCM supplemented with 1 mM NH_4Cl at

30°C in the dark without shaking. Growth was monitored by microscopy, ammonia consumption, and nitrite accumulation as described previously [5, 13]. Late exponential or early stationary phase cultures were transferred to fresh medium (0.25% inoculum), and the purity of cultures was routinely monitored by microscopic inspection and by the absence of bacterial growth in marine broth medium as described previously [5, 13]. The specific growth rates (μ; h^{-1}) were estimated by determining the slope of Ln nitrite concentrations versus time during exponential growth. The generation time (g; h) was calculated as $g = \ln(2)/\mu$. Respiratory activity and NO accumulation of *N. maritimus* cultures were measured with O_2 (Unisense AS, Denmark) and NO (amiNO-600, Innovative Instruments, Sarasota, Florida) microsensors, respectively, in custom-built 35 ml of glass vials as described previously [50].

The genome of *N. maritimus* was initially sequenced in 2007 [51]. Cultures were harvested in May 2011 and June 2016 and stored at -80°C for subsequent genome re-sequencing. Genomes of evolved cultures were sequenced to > 50× coverage on Illumina MiSeq platform using sequenced runs of 2 × 250 PE reads. Raw reads were trimmed and filtered by Trimmomatic (version 0.36) to remove low-quality reads [15]. The high-quality reads were mapped to the reference *N. maritimus* published genome using Bowtie2 (version 2.3.4) [52]. The PCR duplicates of sequence reads were identified and removed by Samtools (version 1.6) [53]. Variant calling for SNP and INDEL was searched by GATK (version 3.8) [54] and Samtools [53]. Variant results were then summarized and filtered by GATK [54].

Distribution and diversity of marine AOA genotypic groups and functional genes in the global ocean

Twenty-five marine AOA species genomes were clustered in 7 genotypic groups to represent populations from distinct phylogenetic lineages and ecological habitats. To investigate the overall distribution of these genotypic groups in the upper ocean (< 1,000 m), competitive fragment recruitment was conducted to determine the relative recruitment to available marine AOA species genomes in GOS and *Tara* Oceans metagenomic databases. In addition, the vertical distribution of these genotypic groups from epi- to hadopelagic waters was determined by competitive fragment recruitment analysis using metagenomic datasets of the North Pacific HOT time-series station (125–4,000 m), Northeast Pacific Ocean (2,000 m), the Yap Trench of the western Pacific (5,000–5,700 m), and the Mariana Trench waters (2,000–8,000 m). Briefly, metagenomic sequences were searched via BLASTP (version 2.2.28+) with an E-value of $\leq 1 \times e^{-10}$ and an identity of ≥80% against an in-house marine AOA species genome database [55]. The recruited amino acid sequences were further classified into seven marine AOA genotypic groups.

To calculate the relative abundance of functional genes in marine AOA natural populations, we first trimmed the raw sequencing reads of *Tara* Oceans and deep ocean metagenomic samples by Trimmomatic (version 0.36) [15]. The trimmed reads were aligned via DIAMOND (version 0.9.24) [56] to the datasets of marine AOA functional genes (including *amoA*, *amt*, *pstB*, *pit*, *mpnS*, and *ureC*) found in available species genomes, MAGs and SAGs. The average fraction of marine AOA populations that possess *pstB*, *pit*, *mpnS*, and *ureC* genes was estimated by calculating the ratio of length-normalized counts for reads that mapped to these accessory functional genes with the criterion of E-value $\leq 1 \times e^{-10}$, reads sequence identity ≥ 80%, and reads length ≥ 100 bp divided by reads that mapped to AOA *amoA* genes, assuming each marine

AOA cell contains one copy of the *amoA* gene (Table S4). The counts of metagenome reads that mapped to AOA high-affinity and low-affinity *amt* genes from environmental samples were calculated using HTSeq (version 0.9.1) and normalized by gene length [57].

To investigate the relative distribution of two homologous genes, A-type and V-type AOA *atpA* throughout the water column, we first mapped the trimmed metagenomic reads to the assembled scaffolds of metagenomic samples collected from epi- to hadopelagic waters using Bowtie2 (version 2.3.4) [52]. The metagenomic sequences of A-type and V-type AOA *atpA* genes were searched via BLASTP (version 2.2.28+) [55] with an E-value of $\leq 1 \times e^{-10}$ and an identity of $\geq$ 80% against an in-house marine AOA species genome database containing *atpA* genes found in available AOA species genomes, MAGs and SAGs. The counts of metagenome reads that mapped to AOA A-*atpA* and V-*atpA* genes from environmental samples were calculated using HTSeq (version 0.9.1) and normalized by gene length [57].

To gain insight into the global diversity of marine AOA *mpnS, atpA*, and *pstB* genes, we compiled a collection of representative sequences of these genes from the *Tara* Oceans [58] and ALOHA [59] gene catalogs. For this purpose, we compiled *mpnS, atpA*, and *pstB* sequences from reference culture genomes, MAGs, and SAGs, aligned them using Clustal Omega [60] and constructed HMM profiles using HMMbuild [61]. These HMM profiles were used to screen the *Tara* Oceans and ALOHA gene catalogs using HMMsearch [61] using E-value of $\leq 1 \times e^{-10}$. These candidate genes were then length filtered (only genes > 200 bp length passed this filter), and initial phylogenetic trees including gene sequences from reference genomes were constructed using the ETE toolkit [62] (workflow: standard_trimmed_fasttree). Outlier

sequences were manually filtered using the phylogenetic trees as guidance and new trees were generated using the same workflow. The phylogenetic trees combining reference sequences and environmental sequences were then visualized using iTOL [63].

Data and materials availability: AOA genomes and metagenomes sequence data are available in the NCBI, JGI, BIGD, or DDBJ databases, and their accession numbers are listed in Table S6. All other data products associated with this study are available from the corresponding authors upon request.

References

1. Widderich N, Czech L, Elling FJ, Könneke M, Stoveken N, Pittelkow M, et al. Strangers in the archaeal world: osmostress-responsive biosynthesis of ectoine and hydroxyectoine by the marine thaumarchaeon *Nitrosopumilus maritimus*. Environ Microbiol. 2016;18:1227-1248.
2. Ngugi DK, Blom J, Alam I, Rashid M, Ba-Alawi W, Zhang GS, et al. Comparative genomics reveals adaptations of a halotolerant thaumarchaeon in the interfaces of brine pools in the Red Sea. ISME J. 2015;9:396-411.
3. Kolp S, Pietsch M, Galinski EA,Gutschow M. Compatible solutes as protectants for zymogens against proteolysis. Biochim Biophys Acta. 2006;1764:1234-1242.
4. Hans Jorg K, Georg L,Erwin AG. Industrial production of the cell protectant ectoine: protection mechanisms, processes, and products. Curr Biotechnol. 2014;3:10-25.
5. Qin W, Amin SA, Martens-Habbena W, Walker CB, Urakawa H, Devol AH, et al. Marine ammonia-oxidizing archaeal isolates display obligate mixotrophy and wide ecotypic variation. Proc Natl Acad Sci USA. 2014;111:12504-12509.
6. Bayer B, Vojvoda J, Offre P, Alves RJE, Elisabeth NH, Garcia JAL, et al. Physiological and genomic characterization of two novel marine thaumarchaeal strains indicates niche differentiation. ISME J. 2016;10:1051-1063.
7. Alonso-Saez L, Waller AS, Mende DR, Bakker K, Farnelid H, Yager PL, et al. Role for urea in nitrification by polar marine Archaea. Proc Natl Acad Sci USA. 2012;109:17989-17994.
8. Kitzinger K, Padilla CC, Marchant HK, Hach PF, Herbold CW, Kidane AT, et al. Cyanate and urea are substrates for nitrification by Thaumarchaeota in the marine environment. Nat Microbiol. 2019;4:234-243.
9. Hallam SJ, Mincer TJ, Schleper C, Preston CM, Roberts K, Richardson PM, et al. Pathways of carbon assimilation and ammonia oxidation suggested by environmental genomic analyses of marine Crenarchaeota. PLoS Biol. 2006;4:520-536.

10. Xu MN, Li X, Shi D, Zhang Y, Dai M, Huang T, et al. Coupled effect of substrate and light on assimilation and oxidation of regenerated nitrogen in the euphotic ocean. Limnol Oceanogr. 2019;64.
11. Santoro AE, Saito MA, Goepfert TJ, Lamborg CH, Dupont CL,DiTullio GR. Thaumarchaeal ecotype distributions across the equatorial Pacific Ocean and their potential roles in nitrification and sinking flux attenuation. Limnol Oceanogr. 2017;62:1984-2003.
12. Ahlgren NA, Chen YY, Needham DM, Parada AE, Sachdeva R, Trinh V, et al. Genome and epigenome of a novel marine Thaumarchaeota strain suggest viral infection, phosphorothioation DNA modification and multiple restriction systems. Environ Microbiol. 2017;19:2434-2452.
13. Qin W, Heal KR, Ramdasi R, Kobelt JN, Martens-Habbena W, Bertagnolli AD, et al. *Nitrosopumilus maritimus* gen. nov., sp nov., *Nitrosopumilus cobalaminigenes* sp nov., *Nitrosopumilus oxyclinae* sp nov., and *Nitrosopumilus ureiphilus* sp nov., four marine ammonia-oxidizing archaea of the phylum *Thaumarchaeota*. Int J Syst Evol Microbiol. 2017;67:5067-5079.
14. Chin CS, Alexander DH, Marks P, Klammer AA, Drake J, Heiner C, et al. Nonhybrid, finished microbial genome assemblies from long-read SMRT sequencing data. Nat Methods. 2013;10:563.
15. Bolger AM, Lohse M,Usadel B. Trimmomatic: a flexible trimmer for Illumina sequence data. Bioinformatics. 2014;30:2114-2120.
16. Walker BJ, Abeel T, Shea T, Priest M, Abouelliel A, Sakthikumar S, et al. Pilon: an integrated tool for comprehensive microbial variant detection and genome assembly improvement. PLOS ONE. 2014;9.
17. Tatusova T, DiCuccio M, Badretdin A, Chetvernin V, Nawrocki EP, Zaslavsky L, et al. NCBI prokaryotic genome annotation pipeline. Nucleic Acids Res. 2016;44:6614-6624.
18. French E, Kozlowski JA, Mukherjee M, Bullerjahn G,Bollmann A. Ecophysiological characterization of ammonia-oxidizing archaea and bacteria from freshwater. Appl Environ Microbiol. 2012;78:5773-5780.
19. Cleary B, Brito IL, Huang K, Gevers D, Shea T, Young S, et al. Detection of low-abundance bacterial strains in metagenomic datasets by eigengenome partitioning. Nat Biotechnol. 2015;33:1053.
20. Koren S, Walenz BP, Berlin K, Miller JR, Bergman NH,Phillippy AM. Canu: scalable and accurate long-read assembly via adaptive k-mer weighting and repeat separation. Genome Res. 2017;27:722-736.
21. Urakawa H, Martens-Habbena W,Stahl DA. High abundance of ammonia-oxidizing archaea in coastal waters, determined using a modified DNA extraction method. Appl Environ Microbiol. 2010;76:2129-2135.
22. Garcia JC, Urakawa H, Le VQ, Stein LY, Klotz MG,Nielsen JL. Draft genome sequence of *Nitrosospira* sp. strain APG3, a psychrotolerant ammonia-oxidizing bacterium isolated from sandy lake sediment. Genome Announc. 2013;1:e00930-00913.
23. Overbeek R, Begley T, Butler RM, Choudhuri JV, Chuang HY, Cohoon M, et al. The subsystems approach to genome annotation and its use in the project to annotate 1000 genomes. Nucleic Acids Res. 2005;33:5691-5702.

24. Matsutani N, Nakagawa T, Nakamura K, Takahashi R, Yoshihara K,Tokuyama T. Enrichment of a novel marine ammonia-oxidizing archaeon obtained from sand of an eelgrass zone. Microbes Environ. 2011;26:23-29.
25. Aziz RK, Bartels D, Best AA, DeJongh M, Disz T, Edwards RA, et al. The RAST server: Rapid annotations using subsystems technology. BMC Genom. 2008;9.
26. Tanizawa Y, Fujisawa T,Nakamura Y. DFAST: a flexible prokaryotic genome annotation pipeline for faster genome publication. Bioinformatics. 2018;34:1037-1039.
27. Albertsen M, Hugenholtz P, Skarshewski A, Nielsen KL, Tyson GW,Nielsen PH. Genome sequences of rare, uncultured bacteria obtained by differential coverage binning of multiple metagenomes. Nat Biotechnol. 2013;31:533.
28. Chan CS, Chan KG, Tay YL, Chua YH,Goh KM. Diversity of thermophiles in a Malaysian hot spring determined using 16S rRNA and shotgun metagenome sequencing. Front Microbiol. 2015;6.
29. Zhang XX, Xu W, Liu Y, Cai MW, Luo ZH,Li M. Metagenomics reveals microbial diversity and metabolic potentials of seawater and surface sediment from a hadal biosphere at the Yap Trench. Front Microbiol. 2018;9.
30. Peng Y, Leung HCM, Yiu SM,Chin FYL. IDBA-UD: a de novo assembler for single-cell and metagenomic sequencing data with highly uneven depth. Bioinformatics. 2012;28:1420-1428.
31. Hyatt D, Chen GL, LoCascio PF, Land ML, Larimer FW,Hauser LJ. Prodigal: prokaryotic gene recognition and translation initiation site identification. BMC Bioinformatics. 2010;11.
32. Kang DWD, Froula J, Egan R,Wang Z. MetaBAT, an efficient tool for accurately reconstructing single genomes from complex microbial communities. PeerJ. 2015;3.
33. Liu Y, Zhou ZC, Pan J, Baker BJ, Gu JD,Li M. Comparative genomic inference suggests mixotrophic lifestyle for Thorarchaeota. ISME J. 2018;12:1021-1031.
34. Sieber CMK, Probst AJ, Sharrar A, Thomas BC, Hess M, Tringe SG, et al. Recovery of genomes from metagenomes via a dereplication, aggregation and scoring strategy. Nat Microbiol. 2018;3:836-843.
35. Liu JW, Zheng YF, Lin HY, Wang XC, Li M, Liu Y, et al. Proliferation of hydrocarbon-degrading microbes at the bottom of the Mariana Trench. Microbiome. 2019;7.
36. Haro-Moreno JM, Lopez-Perez M, de la Torre JR, Picazo A, Camacho A,Rodriguez-Valera F. Fine metagenomic profile of the Mediterranean stratified and mixed water columns revealed by assembly and recruitment. Microbiome. 2018;6.
37. Parks DH, Imelfort M, Skennerton CT, Hugenholtz P,Tyson GW. CheckM: assessing the quality of microbial genomes recovered from isolates, single cells, and metagenomes. Genome Res. 2015;25:1043-1055.
38. Tatusov RL, Galperin MY, Natale DA,Koonin EV. The COG database: a tool for genome-scale analysis of protein functions and evolution. Nucleic Acids Res. 2000;28:33-36.
39. Kanehisa M,Goto S. KEGG: Kyoto Encyclopedia of Genes and Genomes. Nucleic Acids Res. 2000;28:27-30.
40. Bowers RM, Kyrpides NC, Stepanauskas R, Harmon-Smith M, Doud D, Reddy TBK, et al. Minimum information about a single amplified genome (MISAG) and a metagenome-assembled genome (MIMAG) of bacteria and archaea. Nat Biotechnol. 2017;35:725-731.

41. Li L, Stoeckert CJ,Roos DS. OrthoMCL: Identification of ortholog groups for eukaryotic genomes. Genome Res. 2003;13:2178-2189.
42. Enright AJ, Van Dongen S,Ouzounis CA. An efficient algorithm for large-scale detection of protein families. Nucleic Acids Res. 2002;30:1575-1584.
43. Kettler GC, Martiny AC, Huang K, Zucker J, Coleman ML, Rodrigue S, et al. Patterns and implications of gene gain and loss in the evolution of *Prochlorococcus*. PLoS Genet. 2007;3:2515-2528.
44. Grote J, Thrash JC, Huggett MJ, Landry ZC, Carini P, Giovannoni SJ, et al. Streamlining and core genome conservation among highly divergent members of the SAR11 clade. Mbio. 2012;3.
45. Katoh K,Standley DM. MAFFT Multiple sequence alignment software version 7: improvements in performance and usability. Mol Biol Evol. 2013;30:772-780.
46. Talavera G,Castresana J. Improvement of phylogenies after removing divergent and ambiguously aligned blocks from protein sequence alignments. Syst Biol. 2007;56:564-577.
47. Abascal F, Zardoya R,Posada D. ProtTest: selection of best-fit models of protein evolution. Bioinformatics. 2005;21:2104-2105.
48. Stamatakis A. RAxML version 8: a tool for phylogenetic analysis and post-analysis of large phylogenies. Bioinformatics. 2014;30:1312-1313.
49. Könneke M, Bernhard AE, de la Torre JR, Walker CB, Waterbury JB,Stahl DA. Isolation of an autotrophic ammonia-oxidizing marine archaeon. Nature. 2005;437:543-546.
50. Martens-Habbena W, Qin W, Horak REA, Urakawa H, Schauer AJ, Moffett JW, et al. The production of nitric oxide by marine ammonia-oxidizing archaea and inhibition of archaeal ammonia oxidation by a nitric oxide scavenger. Environ Microbiol. 2015;17:2261-2274.
51. Walker CB, de la Torre JR, Klotz MG, Urakawa H, Pinel N, Arp DJ, et al. *Nitrosopumilus maritimus* genome reveals unique mechanisms for nitrification and autotrophy in globally distributed marine crenarchaea. Proc Natl Acad Sci USA. 2010;107:8818-8823.
52. Langmead B,Salzberg SL. Fast gapped-read alignment with Bowtie 2. Nat Methods. 2012;9:357-U354.
53. Li H, Handsaker B, Wysoker A, Fennell T, Ruan J, Homer N, et al. The sequence alignment/map format and SAMtools. Bioinformatics. 2009;25:2078-2079.
54. DePristo MA, Banks E, Poplin R, Garimella KV, Maguire JR, Hartl C, et al. A framework for variation discovery and genotyping using next-generation DNA sequencing data. Nat Genet. 2011;43:491-498.
55. Camacho C, Coulouris G, Avagyan V, Ma N, Papadopoulos J, Bealer K, et al. BLAST plus : architecture and applications. BMC Bioinformatics. 2009;10.
56. Buchfink B, Xie C,Huson DH. Fast and sensitive protein alignment using DIAMOND. Nat Methods. 2015;12:59-60.
57. Anders S, Pyl PT,Huber W. HTSeq-a Python framework to work with high-throughput sequencing data. Bioinformatics. 2015;31:166-169.
58. Sunagawa S, Coelho LP, Chaffron S, Kultima JR, Labadie K, Salazar G, et al. Structure and function of the global ocean microbiome. Science. 2015;348.

59. Mende DR, Bryant JA, Aylward FO, Eppley JM, Nielsen T, Karl DM, et al. Environmental drivers of a microbial genomic transition zone in the ocean's interior. Nat Microbiol. 2017;2:1367-1373.
60. Sievers F, Wilm A, Dineen D, Gibson TJ, Karplus K, Li WZ, et al. Fast, scalable generation of high-quality protein multiple sequence alignments using Clustal Omega. Mol Syst Biol. 2011;7.
61. Eddy SR. Accelerated Profile HMM Searches. PLoS Comput Biol. 2011;7.
62. Huerta-Cepas J, Serra F,Bork P. ETE 3: reconstruction, analysis, and visualization of phylogenomic data. Mol Biol Evol. 2016;33:1635-1638.
63. Letunic I,Bork P. Interactive Tree Of Life (iTOL) v4: recent updates and new developments. Nucleic Acids Res. 2019;47:W256-W259.

Supplementary Tables

Table S1. Summary information of AOA species genomes and MAGs as well as the genomes of other relevant microbial groups.

Table S2. The 71 genes shared among 44 and 65 ammonia-oxidizing thaumarchaeotal genomes that were used to construct the concatenated phylogenomic trees in Figure 2 and Figure S1, respectively.

Table S3. Average nucleotide identity (ANI) between two AOA genomes and fraction of genes shared between the two genomes.

Table S4. Distribution of major metabolic pathway genes in the analyzed AOA genomes. Numerals indicate the numbers of homologs in each genome. Darker shades of red represent an increasing number of homologs, and white represents the lack of respective COGs in the genome.

Table S5. Description of the non-synonymous and synonymous mutations observed during continuous culturing of *Nitrosopumilus maritimus*.

Table S6. The list of AOA genomes and metagenomes used in this study. Sequence data are available through accession numbers in NCBI, JGI, BIGD, or DDBJ databases.
