# Supplementary material for: Alternative strategies of nutrient acquisition and energy conservation map to the biogeography of marine ammonia-oxidizing archaea
Source: ISME J. 2020 Jul 7;14(10):2595–609. doi: 10.1038/s41396-020-0710-7 (PMC7490402; doi:10.1038/s41396-020-0710-7)
Supplement: Supplementary file 2 — Supplementary Figures [file 41396_2020_710_MOESM2_ESM.pdf]

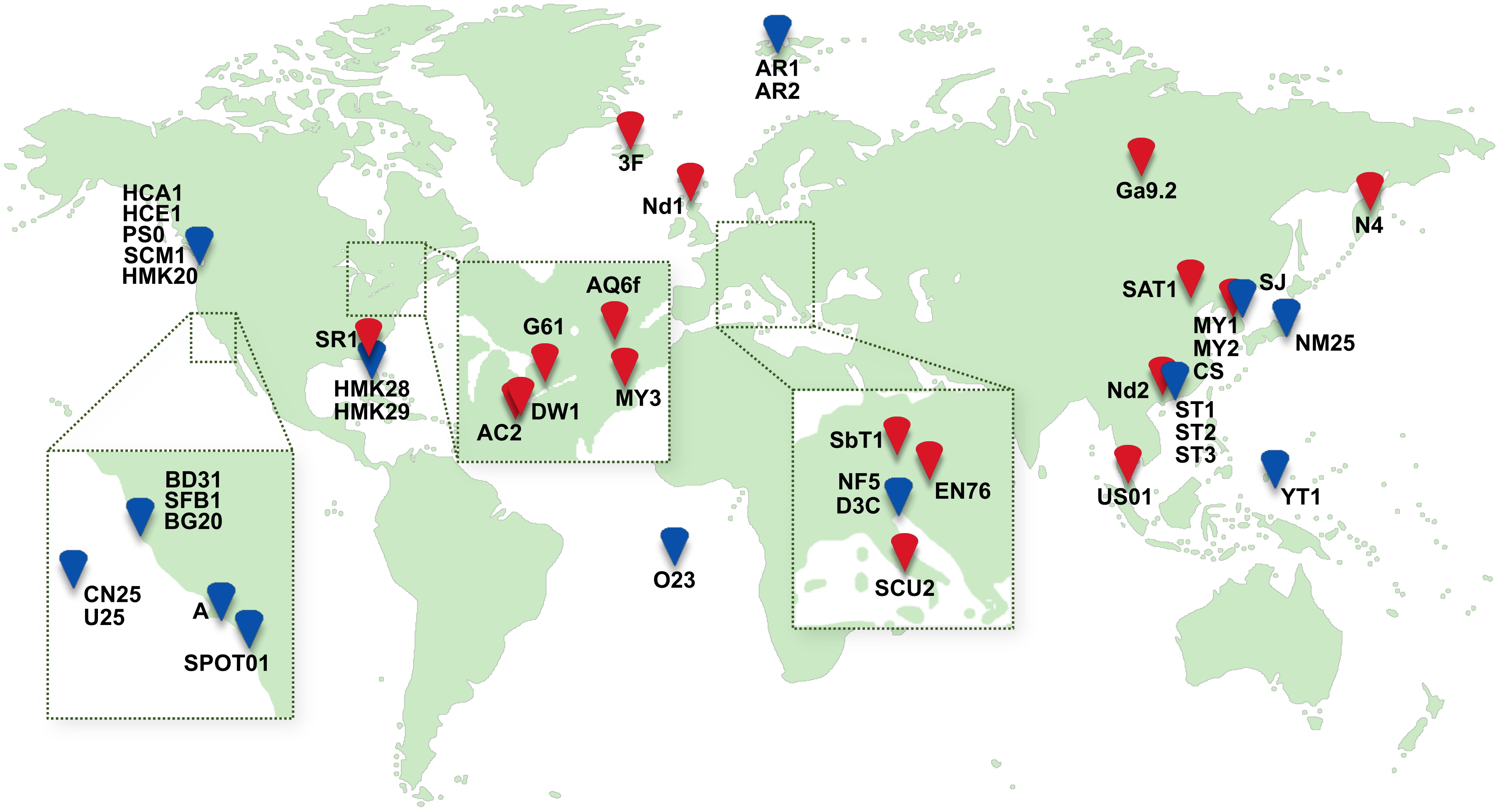

Figure S1. The geographic origin of the 44 AOA species described in this study. The blue and red pins indicate the locations of marine and terrestrial AOA species, respectively.

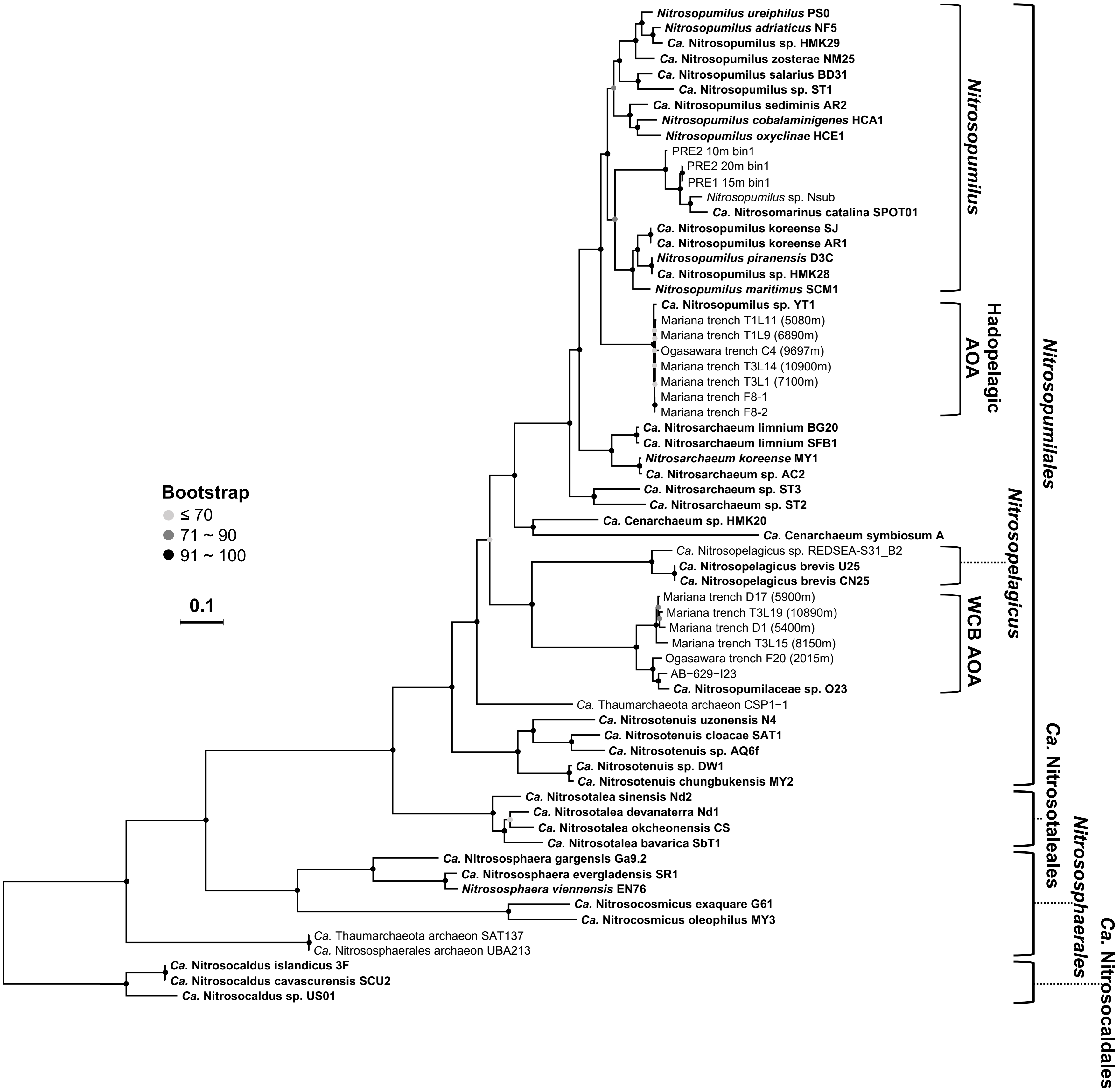

Figure S2. Phylogeny of 65 AOA genomes based on concatenated sequences of 71 single-copy core genes (Table S2). The 44 culture genomes and MAGs shown in Figure 1 and used for comparative genomic analysis are highlighted in bold. Confidence values are on the basis of 100 bootstrap replications. The scale bar represents 10% estimated sequence divergence.

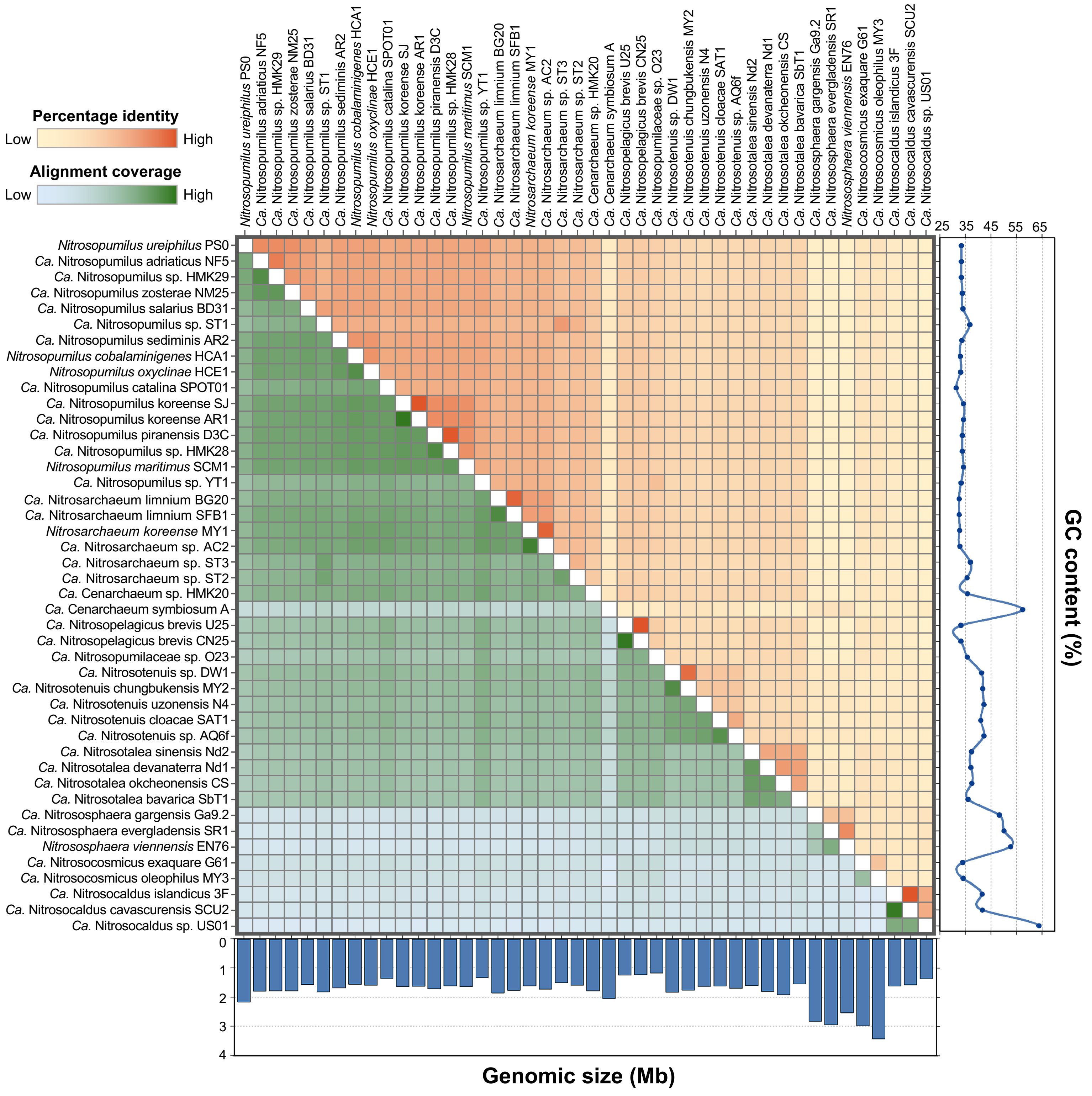

Figure S3. Heatmap showing the fraction of the total genes in the genome shared between two AOA genomes (bottom left half of the matrix) and the average nucleotide identity (ANI) of the shared genes between the two genomes (upper right half of the matrix). Genome size and GC content (%) are displayed at the bottom and on the side of the matrix, respectively.

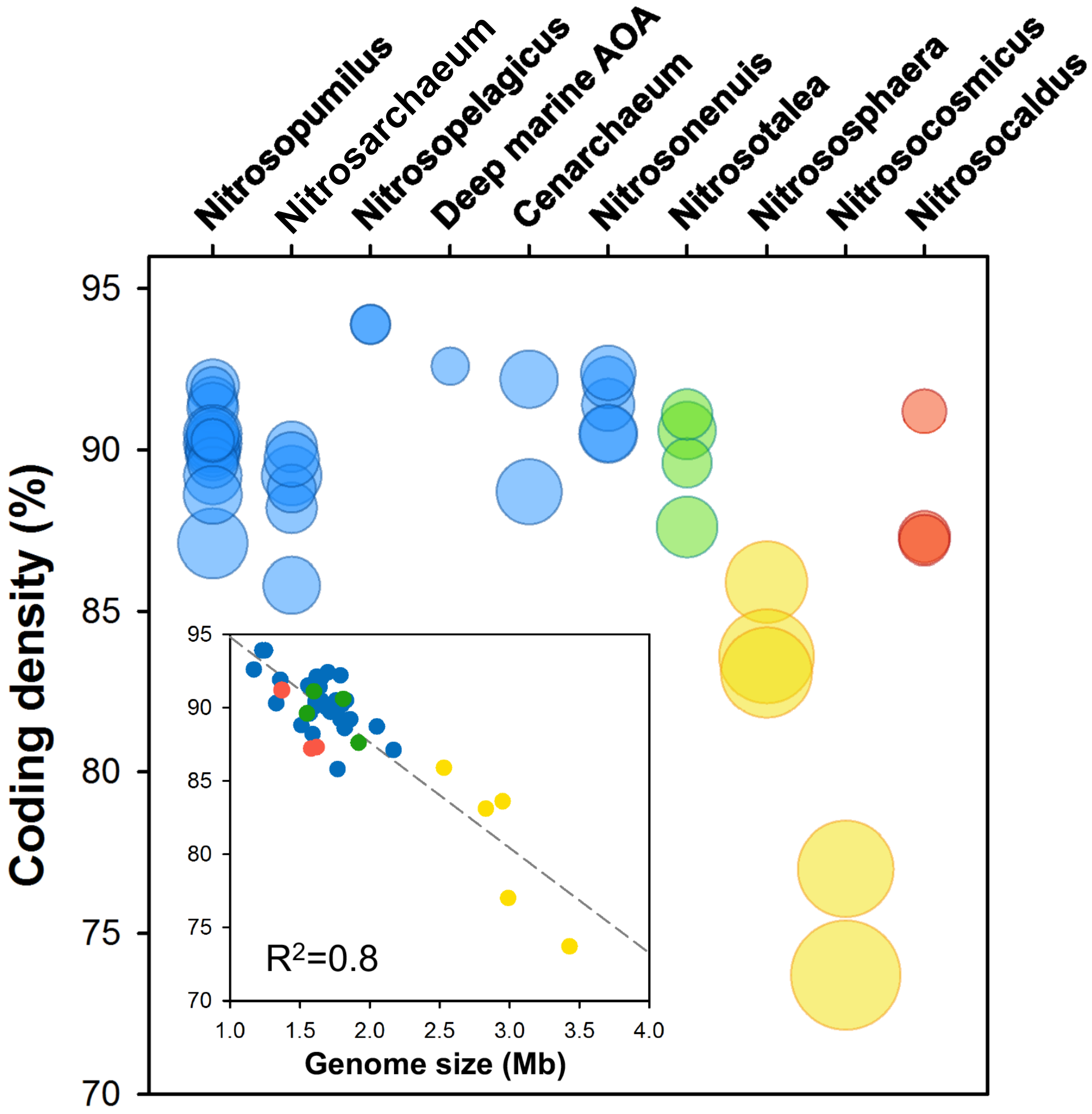

Figure S4. Correlation of genome size and genome coding density of AOA members of the orders *Nitrosopumilales* (blue), *Ca. Nitrosotaleales* (green), *Nitrososphaerales* (yellow), and *Ca. Nitrosocaldales* (red). Bubble size indicates the genome size of AOA species. The linear regression line (inset panel) is y= -7.1891x + 102 (R^2 = 0.8).

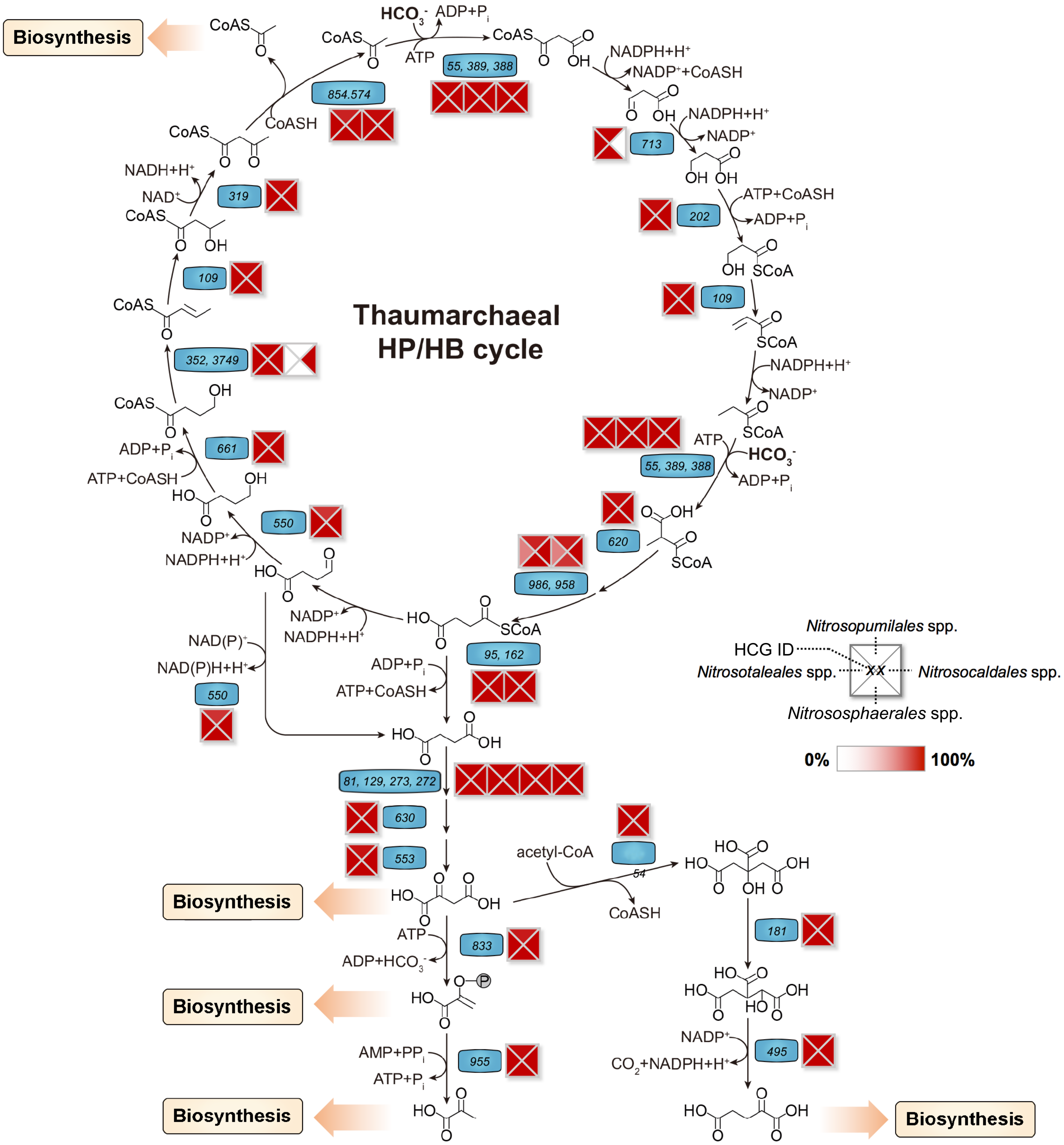

Figure S5. Reconstruction of the thaumarchaeotal 3-HP/4-HB carbon fixation pathway emphasizing the conservation and uniqueness of pathway enzymes of AOA species. Squares indicate the COGs of proteins in AOA species genomes, and COG numbers are inside blue boxes. The figure arrangement and color schemes are the same as those shown in Figure 3.

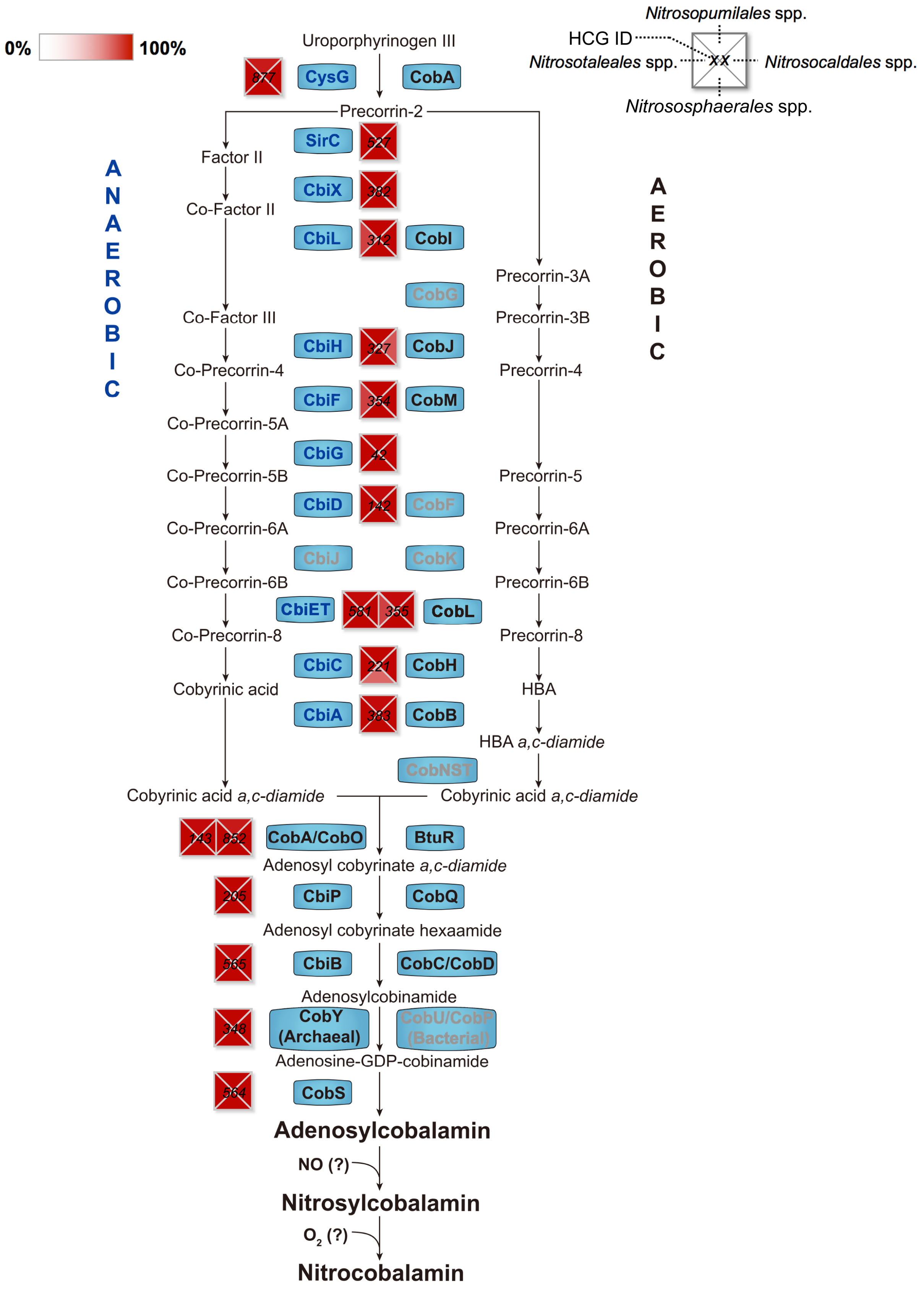

Figure S6. Reconstruction of the thaumarchaeotal cobalamin biosynthetic pathway emphasizing the conservation and uniqueness of pathway enzymes of AOA species. The figure arrangement and color schemes are the same as those shown in Figure 3. Gene names in grey correspond to enzymes not yet identified in thaumarchaeotal genomes.

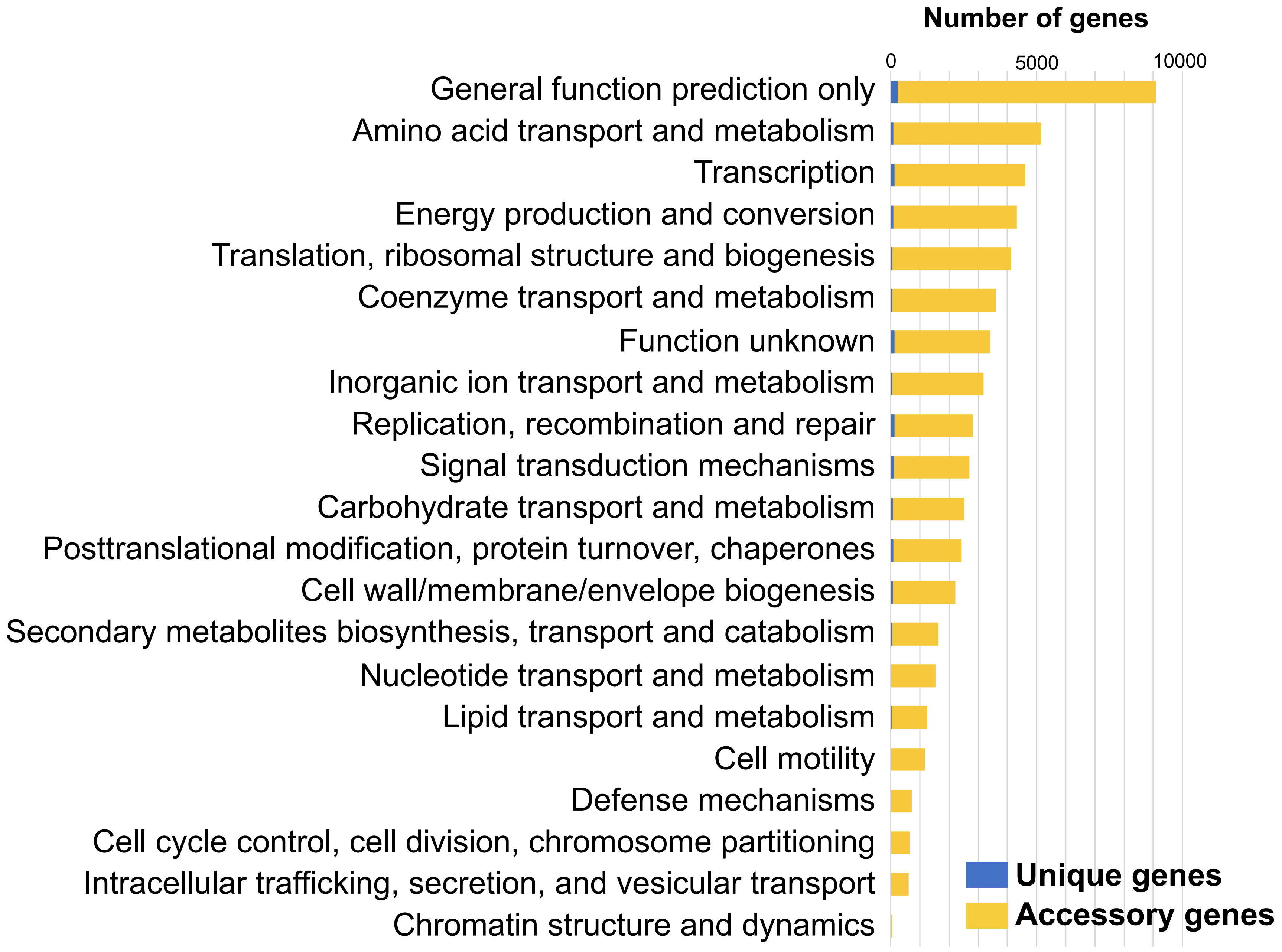

Figure S7. Distribution of the COG functional categories assigned to the accessory and unique genes of AOA pan-genome.

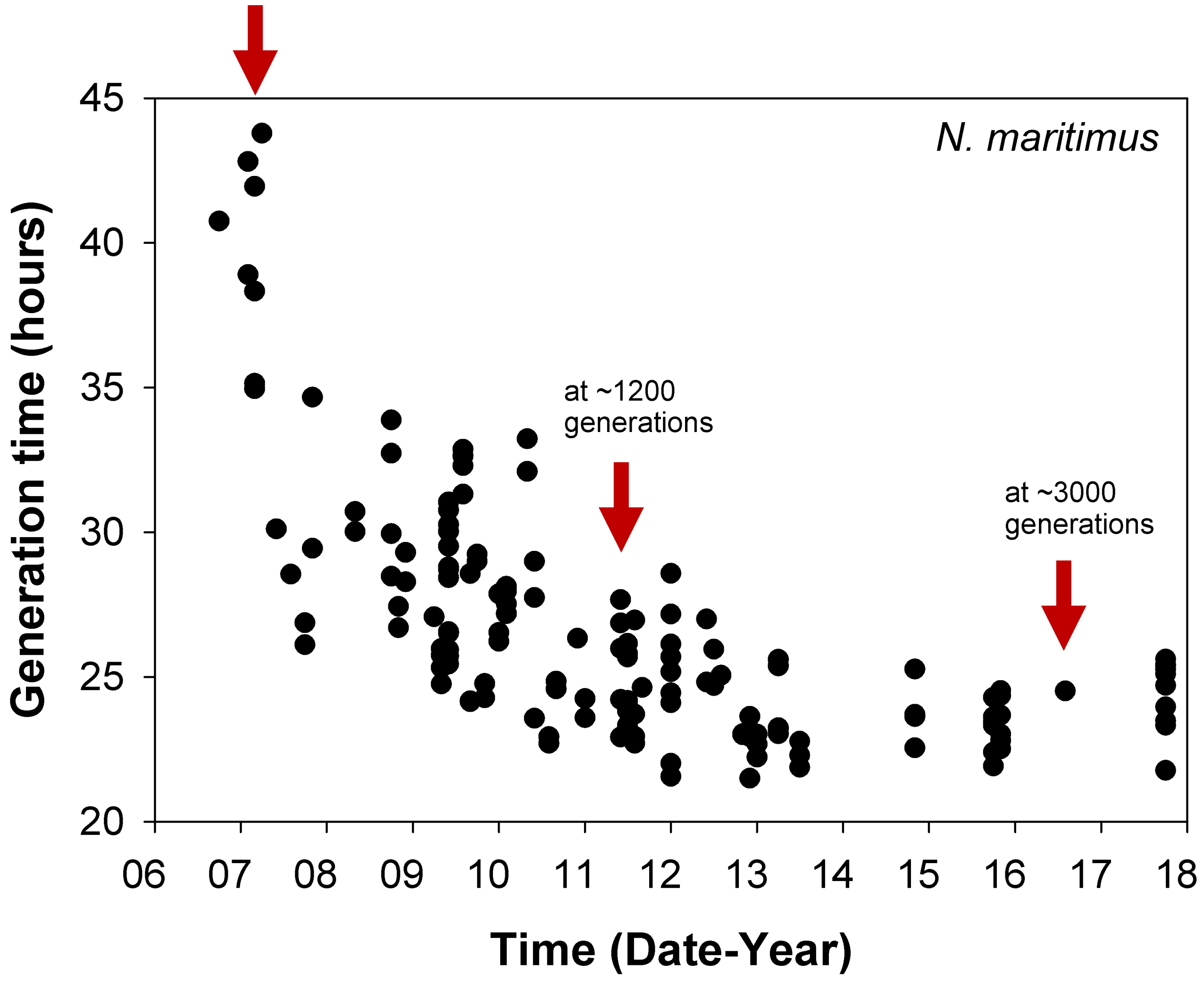

Figure S8. Generation time of *Nitrosopumilus maritimus* during adaptive growth over 11 years of continuous transferring. We measured generation time in the exponential phase of growth. Red arrows indicate that the evolved cultures were collected in 2011 and 2016 and mutations were characterized by genome resequencing compared with the ancestral culture genome sequenced in 2007.

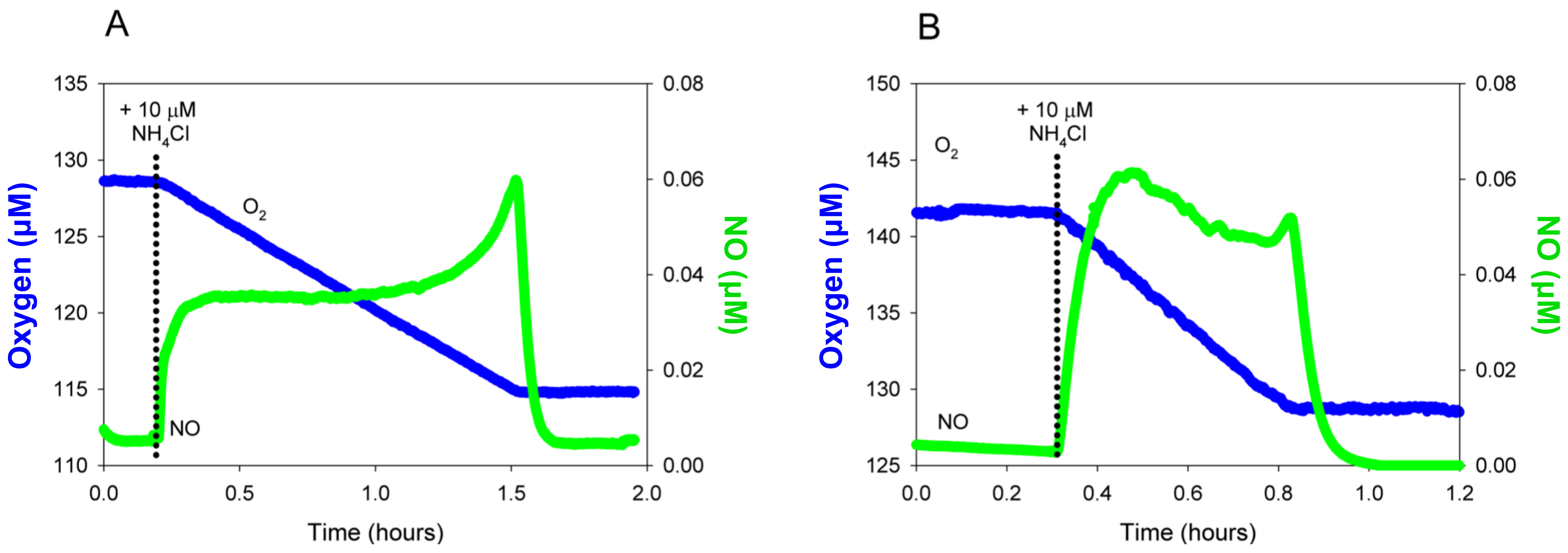

Figure S9. Oxygen uptake and NO accumulation measurements following ammonia addition to ammonia-depleted ancestral (A) and evolved (B) cultures of *Nitrosopumilus maritimus*.

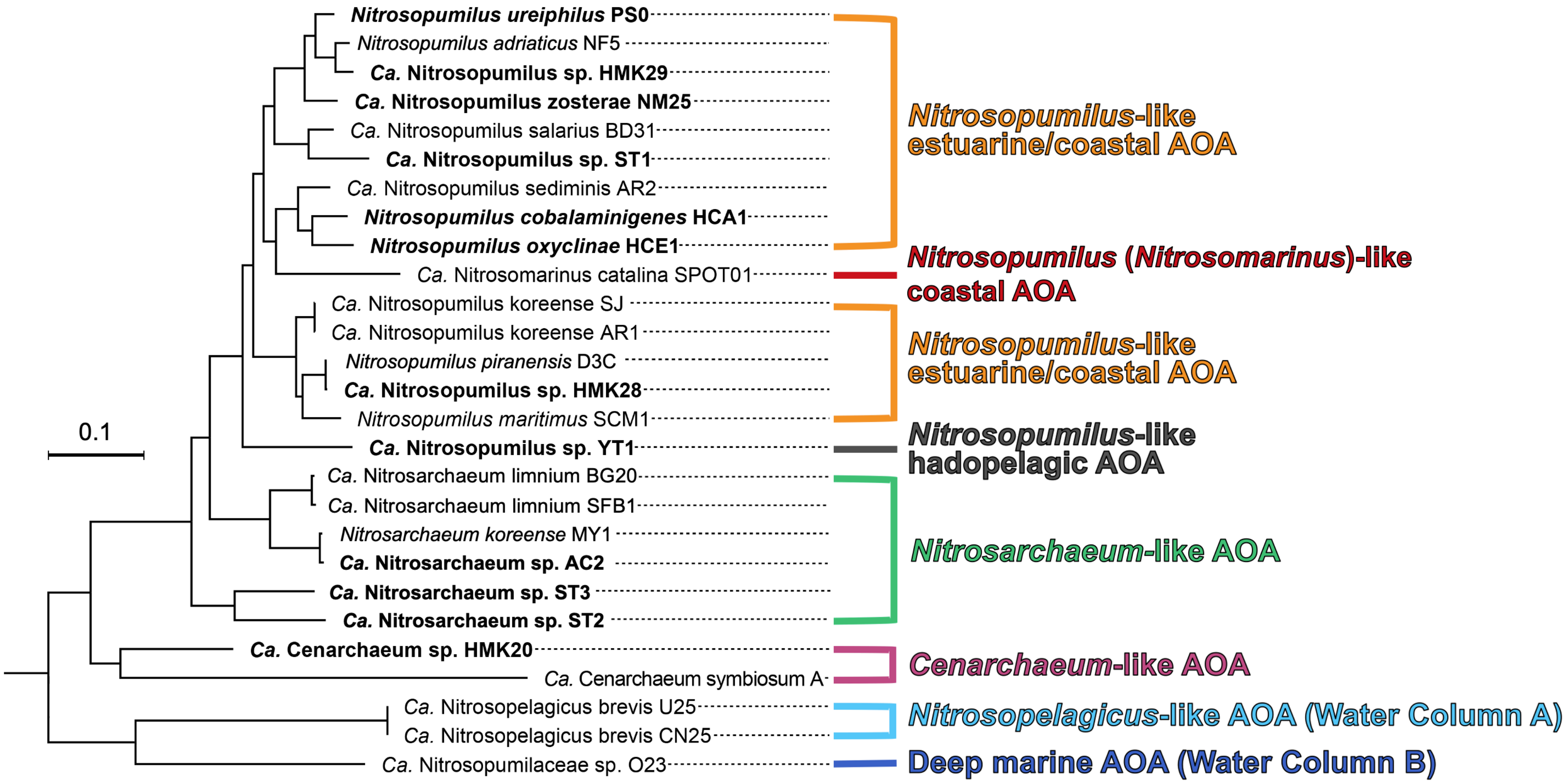

Figure S10. Marine AOA genotypic group assignment based on the position of 25 marine AOA species in the phylogenomic tree and their distinct geographic distributions.

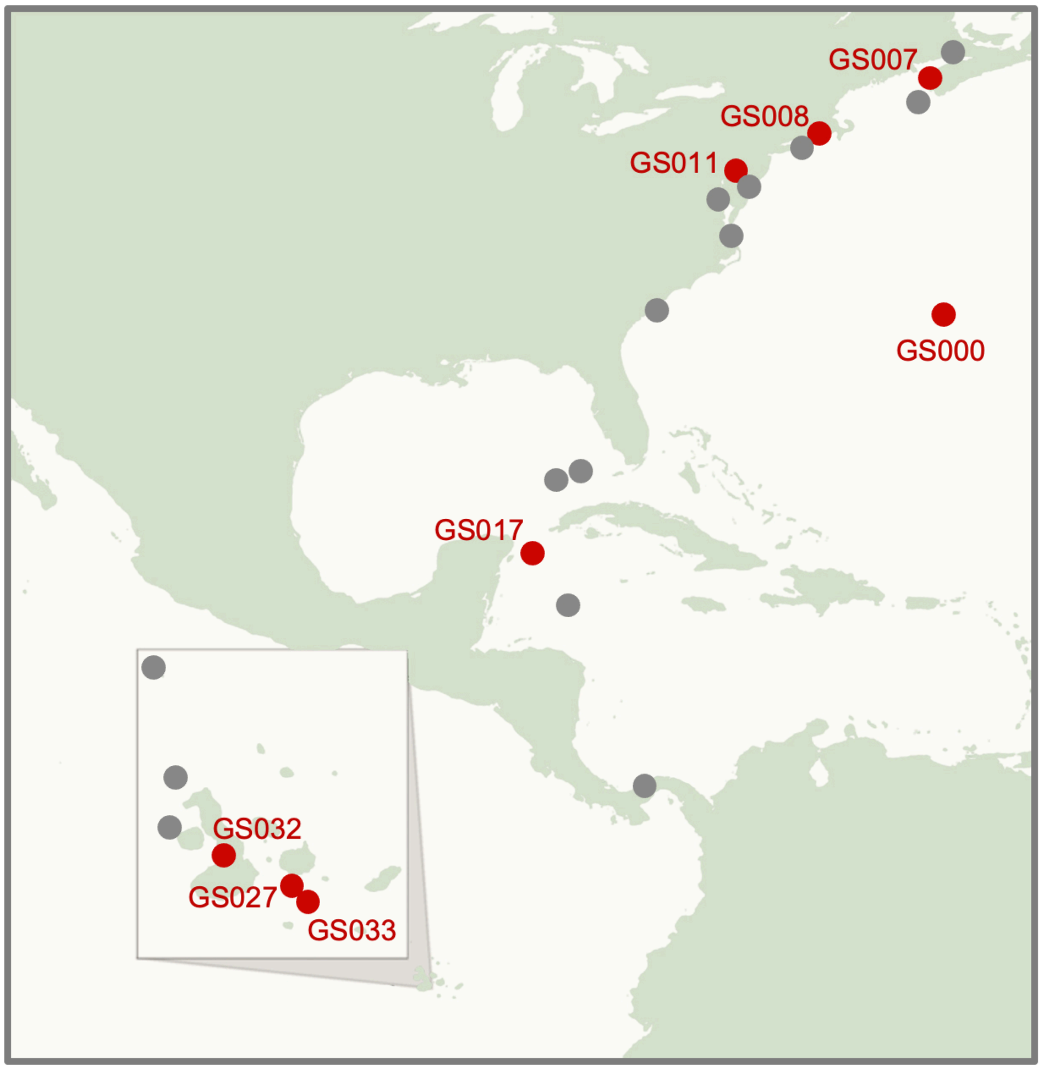

Figure S11. Map depicting from where AOA ectoine/hydroxyectoine biosynthetic genes were recovered in GOS metagenomes. Red dots and grey dots indicate the GOS sampling stations with and without ectoine/hydroxyectoine biosynthetic genes detection, respectively.

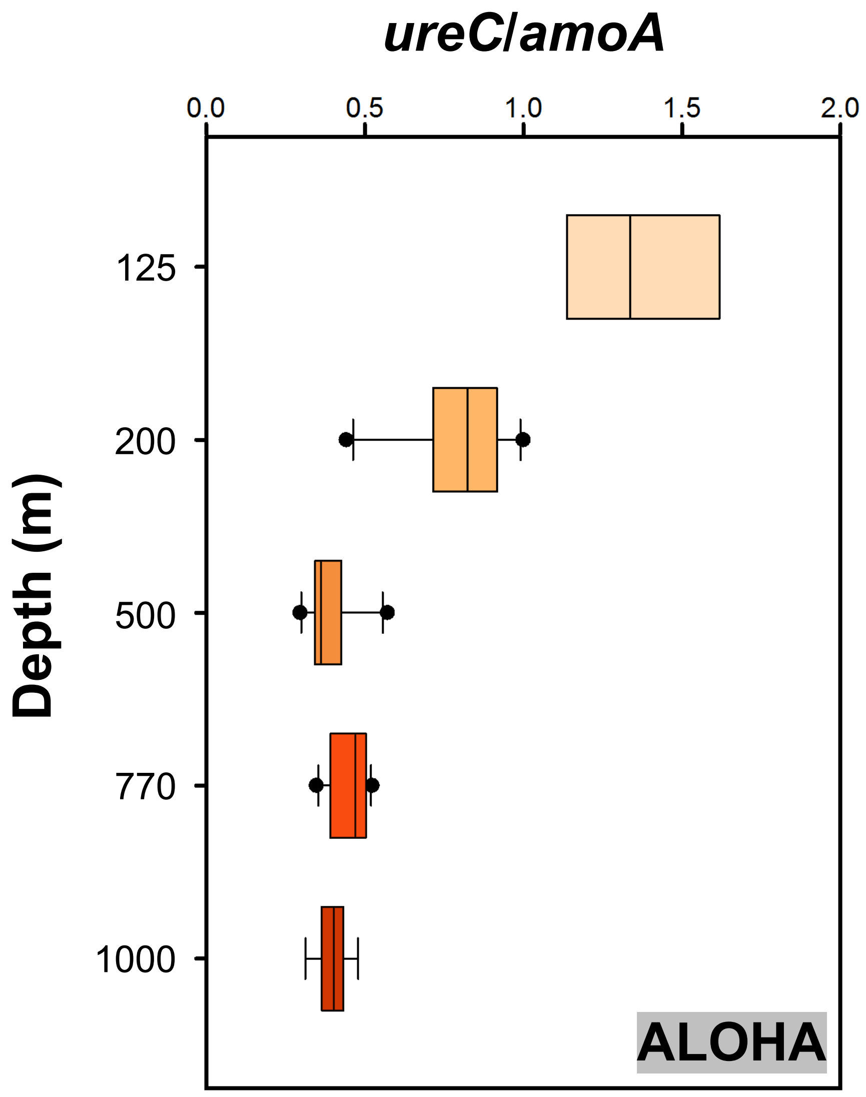

Figure S12. Box-and-whisker plots of the estimated relative abundance of *ureC* genes in marine AOA populations throughout the upper ocean water column at Station ALOHA by metagenomic read recruitment. Metagenomic sequencing data were generated from samples obtained at Station ALOHA during HOT cruises 224 225, 227, 229, 231, 232, 233, 234, 236, 237, and 238. The relative ratio of *ureC* to *amoA* genes was calculated as the ratio of gene length-normalized coverage for the reads that with a minimum length of 100 bp, a maximum E-value of $1 \times e^{-10}$, and a minimum sequence identity of 80% to marine AOA *ureC* and *amoA* genes found in cultures and environmental clones. The high *ureC*:*amoA* ratios over 1.0 may reflect different identity thresholds of between sequence divergence of two genes.

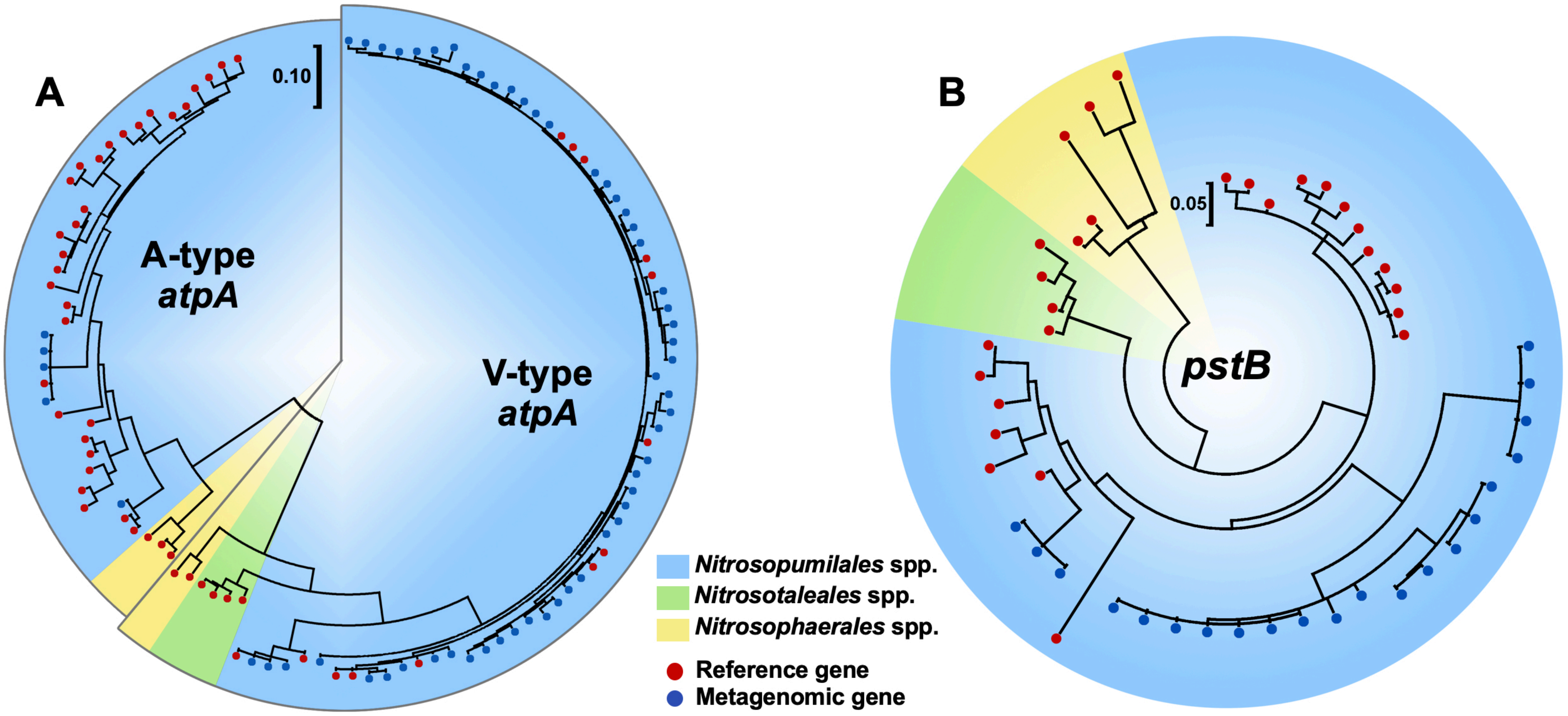

Figure S13. Phylogenetic patterns of AOA *atpA* (encoding the alpha subunit of ATPase) and *pstB* genes. The AOA *atpA* (*A*) and *pstB* (*B*) genes that were recovered from *Tara* Oceans metagenomes are denoted with blue dots at the branch ends. The *atpA* and *pstB* genes of cultured AOA species are denoted with red dots. The blue, green, and yellow shadings represent the *atpA* and *pstB* genes affiliated to the order *Nitrosopumilales*, *Ca*. *Nitrosotalealas*, and *Nitrososphaerales*, respectively.

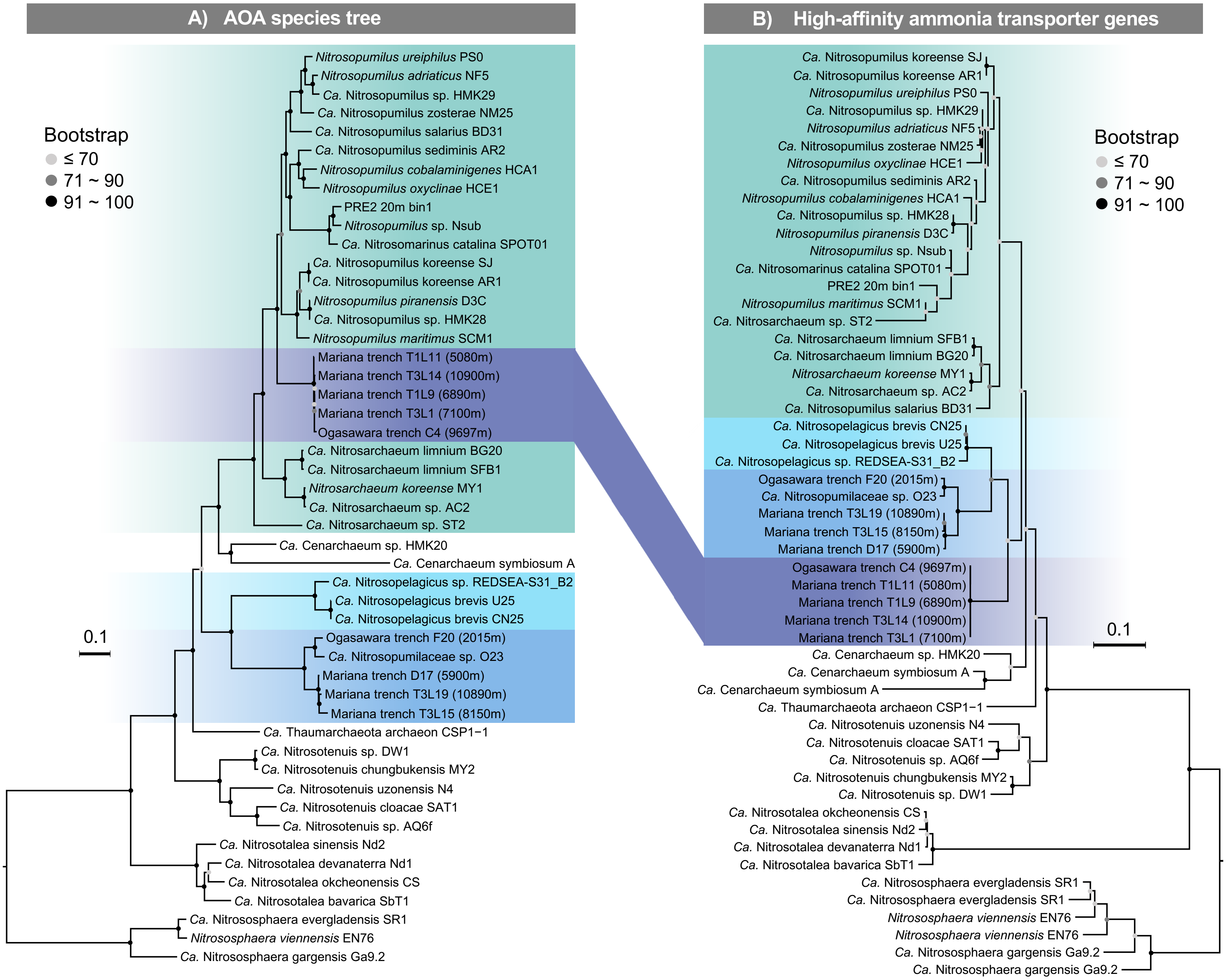

Figure S14. Comparative phylogeny of 71 conserved single-copy genes (Table S2) (A) and high-affinity *amt* genes (B) of marine and soil AOA. The green, light blue, dark blue, and purple shadings highlight genomes and *amt* genes affiliated to the *Nitrosopumilus/Nitrosarchaeum* estuarine/coastal AOA, *Nitrosopelagicus*-like AOA (WCA), deep marine AOA (WCB), and *Nitrosopumilus*-like hadopelagic AOA, respectively. Confidence values are on the basis of 100 bootstrap replications. The scale bar represents 0.1 substitutions per nucleotide position.

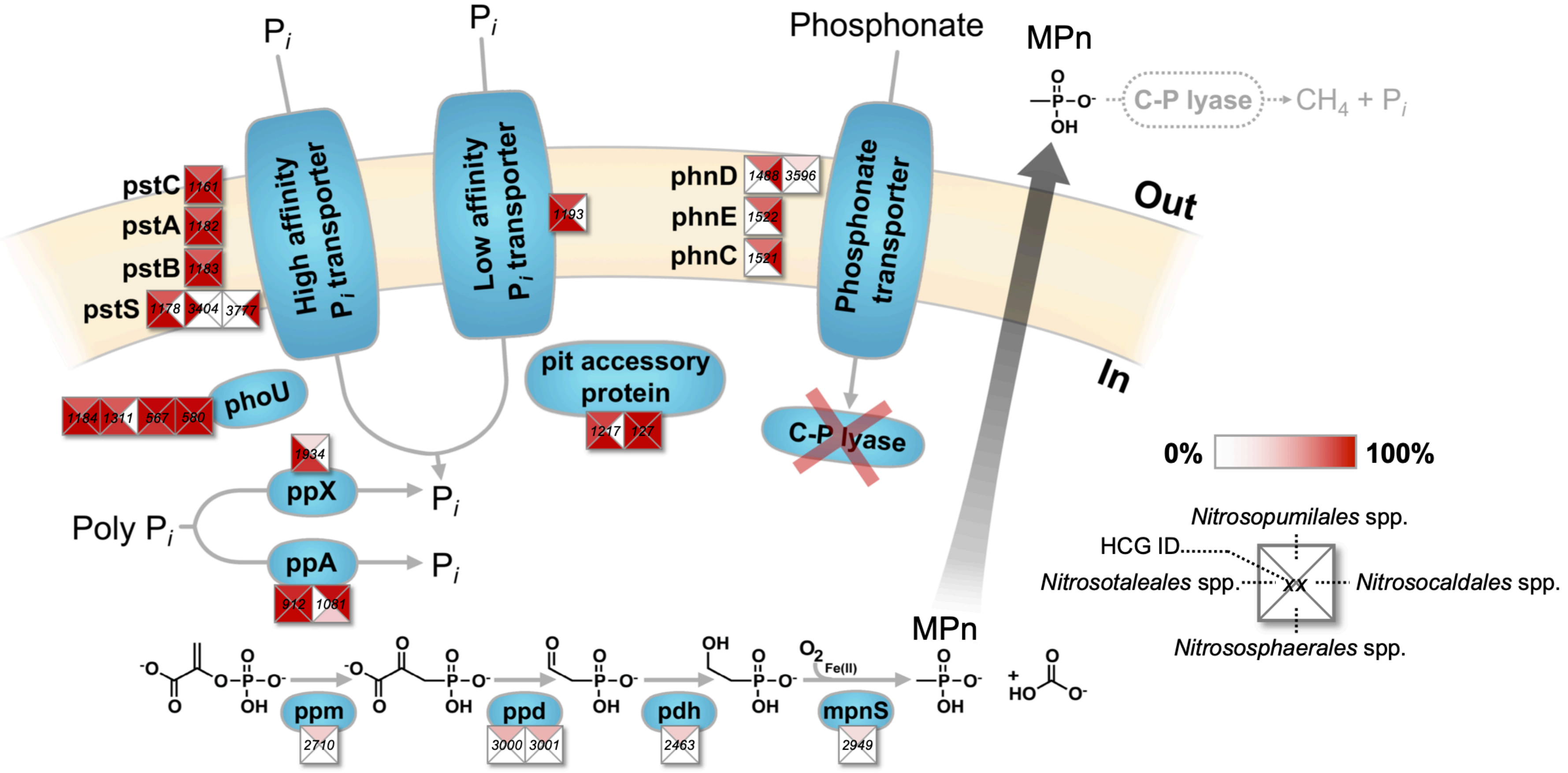

Figure S15. Reconstruction of the proposed pathways of phosphorus acquisition and transformation in AOA emphasizing the conservation and uniqueness of pathway enzymes of AOA species. The figure arrangement and color schemes are the same as those shown in Figure 3. Abbreviations: *pstSCAB*, high-affinity phosphate transport; *phoU*, phosphate uptake regulator; *ppX*, exopolyphosphatase; *ppA*, pyrophosphatase; *phnCDE*, putative phosphonate transport; *ppm*, phosphoenolpyruvate mutase; *ppd*, phosphonopyruvate decarboxylase; *pdh*, phosphonoacetaldehyde dehydrogenase; *mpnS*, methylphosphonic acid synthase; MPn, methylphosphonic acid. Note that no C-P lyase homolog was found in any AOA species. Grey arrow indicates that MPn may be transported out of AOA cells, degraded by other microbes with C-P lyase, which contributes to the methane production in the aerobic ocean.

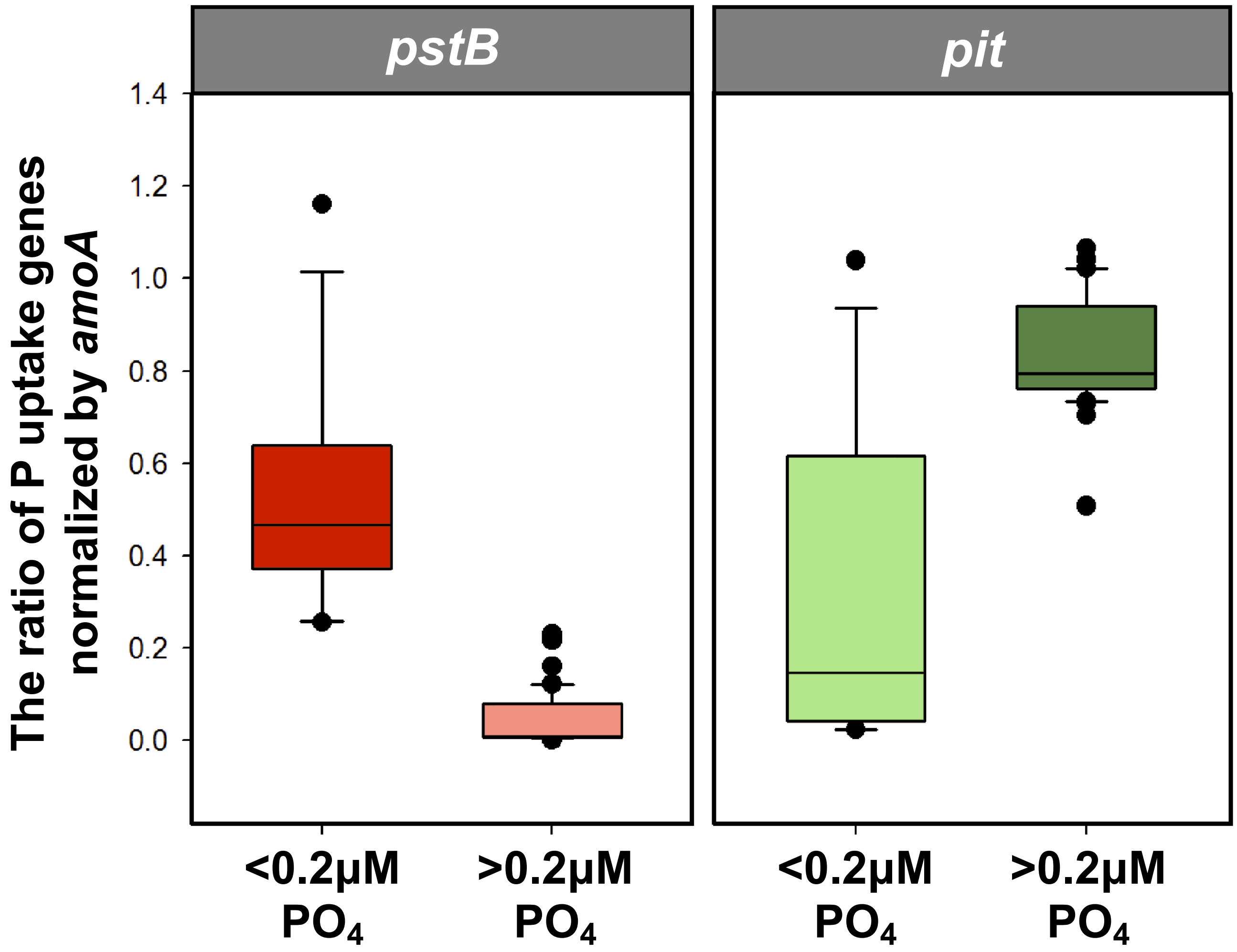

Figure S16. Box-and-whisker plots of the estimated relative abundance of high-affinity (*pstB*) and low-affinity (*pit*) phosphate transporter genes in marine AOA populations from the extremely P-limited (< 200 nM) and relatively P-enriched (0.2 - 3.3 μM) oceanic regions, respectively, by metagenomic recruitment. The relative ratios of *pstB* and *pit* genes to *amoA* genes were calculated as the ratio of gene length-normalized coverage for the *Tara* Oceans metagenomic sequence reads that with a maximum E-value of 1 × e^{-10} and a minimum sequence identity of 80% to the *pstB, pit*, and *amoA* genes found in marine AOA cultured species and MAGs.

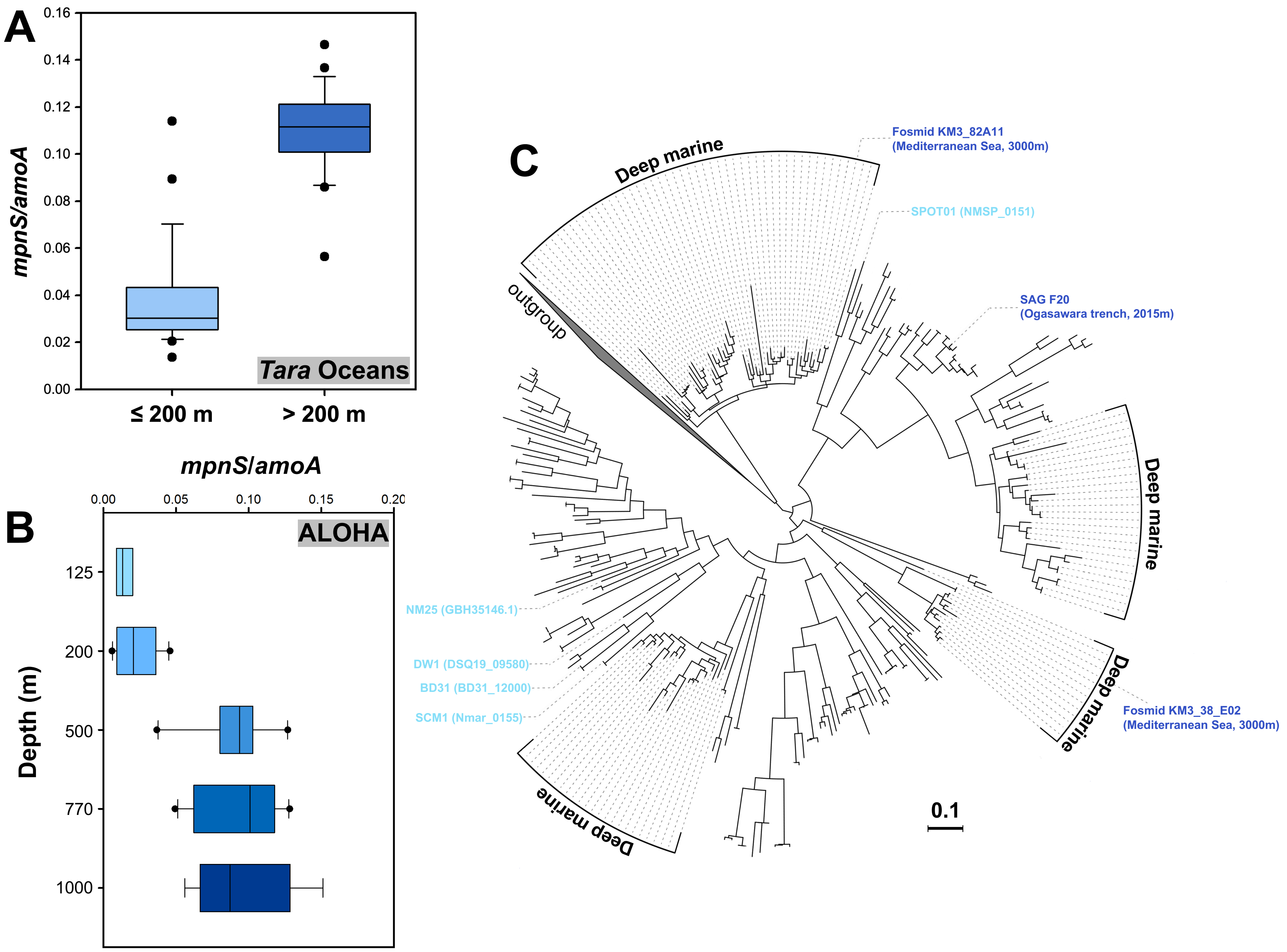

Figure S17. Distribution and diversity of AOA *mpnS* genes in the oceans. (*A*) Box-and-whisker plots of the estimated per-cell *mpnS* gene abundance in marine AOA populations from shallow (≤ 200 m) and deep waters (> 200 m), respectively, at *Tara* Ocean sampling stations by metagenomic recruitment. (*B*) Box-and-whisker plots of the estimated relative abundance of *mpnS* genes in marine AOA populations throughout the upper ocean water column (125-1000 m) at Station ALOHA by metagenomic read recruitment. Metagenomic sequencing data were generated from samples obtained at Station ALOHA during HOT cruises 224, 225, 227, 229, 231, 232, 233, 234, 236, 237, and 238. The per-cell *mpnS* gene abundance was estimated based on the relative ratios of *mpnS* to *amoA* genes by metagenomic read recruitment, assuming one *amoA* gene copy per marine AOA genome. (*C*) Phylogeny of *mpnS* genes retrieved from AOA cultures, fosmids, SAGs, *Tara* Oceans and ALOHA metagenomes. Reference sequences of AOA *mpnS* genes from shallow (≤ 200 m) and deep waters (2000-3000 m) are indicated in light and dark blue text, respectively. "Deep marine" clusters represent the *mpnS* sequences recovered from mesopelagic depths (250-1000 m) at *Tara* Oceans and ALOHA stations. The scale bar represents 0.1 substitutions per nucleotide position.
